# Molecules and Mechanisms to Overcome Oxidative Stress Inducing Cardiovascular Disease in Cancer Patients

**DOI:** 10.3390/life11020105

**Published:** 2021-01-30

**Authors:** Francesco Sabbatino, Valeria Conti, Luigi Liguori, Giovanna Polcaro, Graziamaria Corbi, Valentina Manzo, Vincenzo Tortora, Chiara Carlomagno, Carmine Vecchione, Amelia Filippelli, Stefano Pepe

**Affiliations:** 1 Department of Medicine, Surgery and Dentistry ‘Scuola Medica Salernitana’, University of Salerno, 84081 Baronissi, Italy; vconti@unisa.it (V.C.); gpolcaro@unisa.it (G.P.); vmanzo@unisa.it (V.M.); cvecchione@unisa.it (C.V.); afilippelli@unisa.it (A.F.); spepe@unisa.it (S.P.); 2 Oncology Unit, University Hospital “San Giovanni di Dio e Ruggi D’Aragona”, 84131 Salerno, Italy; 3 Clinical Pharmacology and Pharmacogenetics Unit, University Hospital “San Giovanni di Dio e Ruggi D’Aragona”, 84131 Salerno, Italy; 4 Department of Clinical Medicine and Surgery, University of Naples ‘Federico II’, 80131 Naples, Italy; luigiliguori1992@gmail.com (L.L.); vincenzo.tortor@gmail.com (V.T.); chiara.carlomagno@unina.it (C.C.); 5 Department of Medicine and Health Sciences, University of Molise, 86100 Campobasso, Italy; graziamaria.corbi@unimol.it; 6 Vascular Pathophysiology Unit, I.R.C.C.S. Neuromed, 86077 Pozzilli, Italy

**Keywords:** antioxidative treatment, cardiotoxicity, cardiovascular disease, endothelial dysfunction, oncological treatments, oxidative stress, ROS

## Abstract

Reactive oxygen species (ROS) are molecules involved in signal transduction pathways with both beneficial and detrimental effects on human cells. ROS are generated by many cellular processes including mitochondrial respiration, metabolism and enzymatic activities. In physiological conditions, ROS levels are well-balanced by antioxidative detoxification systems. In contrast, in pathological conditions such as cardiovascular, neurological and cancer diseases, ROS production exceeds the antioxidative detoxification capacity of cells, leading to cellular damages and death. In this review, we will first describe the biology and mechanisms of ROS mediated oxidative stress in cardiovascular disease. Second, we will review the role of oxidative stress mediated by oncological treatments in inducing cardiovascular disease. Lastly, we will discuss the strategies that potentially counteract the oxidative stress in order to fight the onset and progression of cardiovascular disease, including that induced by oncological treatments.

## 1. Introduction

Free radicals, including radical oxygen species (ROS) and reactive nitrogen species (RNS), are products of cellular metabolism involved in cellular redox regulation and physiological functions such as immunity, differentiation, mitogenic response and cytokine production [1,2]. In cellular metabolism ROS, both free radicals such as superoxide (O_2_^−^) and non-radical species such as hydrogen peroxide (H_2_O_2_) display beneficial or detrimental effects since they stimulate stress response and cause oxidative damage and tissue dysfunction. According to the “mitohormesis” theory [3], low or moderate levels of ROS have beneficial effects on biological processes. However high concentration of these molecules generates oxidative stress by overcoming the efficiency of enzymatic and non-enzymatic antioxidant systems [1,2]. The imbalance between ROS production and capability of antioxidant systems results in indiscriminate damage to all cellular constituents including DNA, proteins, and cell membranes. As a result, oxidative stress plays a crucial role in determining cell fate [4,5]. 

The biology of oxidative stress is very complex and multiple mechanisms are involved [6]. However, irrespective of the mechanism, oxidative stress causes the onset of many types of disease such as cardiovascular disease (CVD) and cancer [7,8] as well as modulation of cancer treatment-related outcomes [1,2]. Excessive ROS levels have been linked to tumor initiation, growth and progression [1]. In addition, malignant disease expresses higher levels of ROS production as compared to normal tissues because of increased metabolic rate, oncogene activation and defective vasculature. The latter leads to the generation of hypoxic areas [2]. Lastly, cytotoxicity of chemotherapeutic agents can be mediated by ROS generation leading to induction of apoptosis or necrosis in both normal and cancer cells by exceeding the antioxidant capacity of the tissue [2]. 

In this review, we will first summarize the biology and mechanisms of ROS mediated oxidative stress, underlying the role of ROS in the onset and progression of CVD. Second, we will review oncological treatments which induce CVD, emphasizing the crucial role of oxidative stress in mediating this type of toxicity. Lastly, we will discuss the strategies potentially useful to counteract the oxidative stress in order to fight the onset and progression of CVD, including that induced by oncological treatments.

## 2. Biology and Mechanisms of Oxidative Stress

Mitochondria are intracellular organelles which represent the “powerhouses” of the cells. Their functionality is crucial especially in post-mitotic tissues such as the heart. In heart tissue a huge amount of energy is required. This energy is provided in the form of adenosine triphosphate (ATP) that is synthesized by mitochondria. Mitochondria produces ATP starting from substrates such as fatty acids, glucose and amino acids, in a process called oxidative phosphorylation. The latter occurs in a series of complexes, named electron transfer chain (ETC), that transfers electrons from electron donors to electron acceptors via redox reactions. During these reactions, ROS are continuously produced through an oxidative metabolism [9]. 

Conditions causing mitochondrial dysfunction are associated with an increased ROS production. Mitochondrial dysfunction is determined to a large extent by genomic and mitochondrial DNA instability as well as by epigenetic alterations [10,11]. DNA instability is strictly correlated to several syndromes linked to premature cardiovascular aging [12]. Moreover, impairments in ETC not only affect the ATP synthesis, but compromise several mitochondrial functions including calcium and redox homeostasis, leading to calcium overload and redox balance alteration [13]. In cardiomyocytes, calcium overload leads to cellular damage and death by increasing mitochondrial ROS production [14]. Changes in the redox state homeostasis influence apoptotic events to a variable extent depending on cell types and tissues. Heart and brain are mainly affected with an increased incidence of heart failure and neurodegeneration that are often inter-connected [15,16]. 

Mitochondria produce free radicals from various substrates by utilizing multiple enzymes such as xanthine oxidase and NADH/NADPH oxidases (NOXs). These enzymes, expressed in endothelial cells, cardiomyocytes and other cardiovascular cells, continuously produce ROS. In addition, in presence of various stimuli such as cyclic stretch, inflammation and pharmacological therapy these and other enzymes such as the cytochrome p450s and the peroxidases increase the amount of ROS. As a result, excessive ROS production causes the onset and progression of cardiac dysfunction and CVD [17]. 

Several alterations of CVD have in common endothelial dysfunction, a condition determined by oxidative stress accumulation and decline in nitric oxide (NO) bioavailability. The latter is caused by a decrease of the expression and activity of endothelial NO synthase (eNOS), alteration of cellular signaling with a consequent paucity of eNOS substrates and co-factors, and ROS-mediated NO degradation. NO is inactivated by ROS, mainly O_2_^−^·, in a reaction that is counteracted by the antioxidant enzyme superoxide dismutase (SOD). SOD largely contributes to maintain the delicate NO/O_2_^−^· balance [18]. Alterations in the expression and/or activity of SOD as well as of endogenous antioxidant enzymes such as catalase and glutathione peroxidase (GSH-Px) also cause oxidative stress that contributes to cell and tissue dysfunction [19]. Several studies reported a negative correlation between systemic antioxidant capacity and development of diseases, primarily CVD [7]. 

## 3. Oncologic Treatment Related Cardiotoxicity: The Role of Oxidative Stress

Several anticancer agents are known to cause reversible and/or irreversible cardiomyopathies as well as blood pressure alterations, arrhythmias, vasculitis and other types of CVD [20]. In addition, cardiovascular toxicity represents a dose limiting factor for many anticancer treatments [21]. Cytotoxicity from anticancer agents in both cancer and normal cells can be mediated by both a direct cytotoxic effect and an increased ROS production. As already mentioned, ROS production is mitigated by antioxidant enzymes. Several lines of evidence suggest that cancer tissues upregulate the expression of antioxidant enzymes as a protective mechanism to counteract an increased oxidative stress in an adaptive manner [21]. This protective mechanism seems to be less effective in normal tissues including the heart as compared to cancer cells. As a result, cardiac cells are more susceptible to cell alterations as compared to cancer cells. Even more the specific susceptibility of the cardiac cells to the oxidative stress induced by anticancer agents is also affected by the relatively low levels of antioxidant enzymes [22]. 

Understanding the mechanisms of drug-related cardiotoxicity becomes crucial to design novel strategies in order to promote an effective cardio protection, without compromising the efficacy of anticancer treatments. Due to ethical reasons and difficulty in obtaining patients’ myocardial biopsies most of the available data essentially comes from in vivo and in vitro experimental models. In vivo studies, involving rodents and non-rodents species, can mimic chronic cardiotoxicity observed in clinical practice as they allow repeated drug administrations and analysis of tissues for histopathologic alterations [23]. Anthracyclines-related cardiotoxicity has been extensively studied in C57Bl/6 and BALB/C mice which are the most characterized strains used in the laboratory [24]. Afterward, a variety of oncological agents (both cytotoxic and targeted compounds) has also been found to induce cardiovascular alterations during preclinical studies by using mouse, rat and rabbit models [23]. 

In the context of oxidative stress-related cardiotoxicity, animal models also allow the cardioprotective effects of several substances to be discovered. For instance, Qi et al. demonstrated that cardamonin, a flavonoid isolated from several herbs, alleviated doxorubicin-induced cardiotoxicity in C57BL/6 J mice. Specifically, cardamonin treatment was effective in counteracting oxidative stress in the hearts of doxorubicin-treated mice by increasing antioxidant and decreasing oxidant species [25]. Chakrabort et al. demonstrated that an atypical G protein Gb5 was responsible to initiate multiple pathologic signaling pathways involved in heart damage associated not only to anthracycline but also taxane, and fluoropyrimidine treatment. In vascular muscle cells treated with these chemotherapeutics, scavenging of ROS reduced apoptosis in a Gb5-dependent manner and Gb5 knockdown in mice, reduced hallmarks of the failing heart including proinflammatory cytokines and profibrotic factors [26]. 

ROS accumulation affects cardiac homeostasis through the alteration of different cellular components. Therefore, in vitro studies have been carried out in different cell types including endothelial cells of the endocardium and coronary vessels, smooth muscle cells, cardiac fibroblasts and cells with progenitor characteristics [27].

The use of cell cultures allows human heart physiology to be mimicked, and offers the advantages of gene manipulation, high throughput, ease of use, and low costs [22]. Recently human pluripotent stem cell (hPSC)-derived cardiomyocytes have been recognized as a helpful tool for assessing cardiotoxicity of anticancer therapeutics. Through such an in vitro model, it has been shown that ROS production increases already 24 h after exposure to doxorubicin at concentrations that are significantly lower when compared to those measurable in patients’ serum levels after a single injection. Notably, repeated doxorubicin exposure irreversibly reduces ATP levels thereby causing mitochondrial dysfunction which cannot be restored after drug removal. 

The hPSC-based in vitro models has been successfully used also to assess cardiac safety of other classes of anti-cancer drugs such as signaling inhibitors and also to compare the related cardiotoxic effects to each other. It has been demonstrated that sunitinib, crizotinib, nilotinib, and erlotinib cause an increase of ROS generation and apoptosis with consequent induction of arrhythmic beating [28].

Unfortunately, the results obtained in animal models are still difficult to fully translate to humans. One of the most important reasons is the potential development of spontaneous cardiovascular alterations in non-treated animals which represents an important confounder for cardiovascular safety assessments. Moreover, the animals usually used in preclinical studies are young and healthy. This limits the occurrence of the aforementioned confounder and does not allow the experimental data to be efficiently translated into older patients [23]. Undoubtedly, also in vitro models have several limitations mainly the lack of multicellular interactions and tissue organization resembling physiology. For these reasons, despite both in vitro and in vivo models represent precious tools to clarify the mechanisms of cardiotoxicity associated to oncological agents, it is crucial to integrate pre-clinical and clinical data [29]. 

### 3.1. Anthracyclines

Anthracyclines are potent cytotoxic drugs that inhibit cancer growth through multiple mechanisms including promotion of ROS. ROS destroy nucleic acids and other biomolecules, inhibiting DNA synthesis as well as topoisomerase II activity [30]. Anthracyclines are currently approved for the treatment of both hematological and solid malignancies. Doxorubicin, daunorubicin, epirubicin and idarubicin are the most common anthracyclines utilized in oncology. Doxorubicin and daunorubicin were the first to be used in clinical practice. Epirubicin, a stereoisomer of doxorubicin, has an increased volume of distribution and longer half-life as compared to doxorubicin. Idarubicin, a derivative of daunorubicin, is more lipophilic and has a higher cellular uptake as compared to daunorubicin [31]. 

The clinical use of anthracyclines is often limited by their related cardiotoxicity, well described by a rich body of literature [31]. The incidence of cardiotoxicity during anthracycline administration ranges from 0.9% to 26.0%. This incidence depends by the cumulative dose of anthracycline used and patient characteristics such as cardiovascular risk and age. Use of anthracyclines may lead to acute, chronic or late-onset cardiotoxicity. Acute cardiotoxicity includes arrhythmias and elevation of levels of brain natriuretic peptide and troponin. These adverse events are reversible in a week after discontinuation of treatment and the chemotherapy agent may be resumed later. Chronic cardiotoxicity results in cardiomyopathy and usually occurs within one year of treatment. Late-onset cardiotoxicity occurs from months to years after treatment. Patients present a progressive decline in ejection fraction that leads to decompensation, valvular damage or worse arrhythmias [31,32]. 

**Figure 1:**
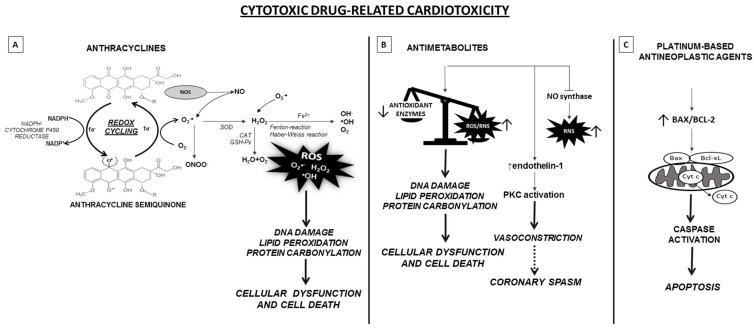
Schematic representation of the molecular mechanisms underlying cytotoxic drugs-related cardiotoxicity. (**A**) Anthracyclines undergo redox cycling catalyzed by NADPH-Cytochrome P-450 reductase. The one-electron (1e^−^) reduction of the quinone compound leads to the formation of semiquinone intermediate, that in presence of molecular oxygen auto-oxides generating the parent anthracycline and O_2_^−^∙. O_2_^−^∙ is either transformed to RNS or dismutated to H_2_O_2_ by SOD. Subsequently, H_2_O_2_ is eliminated by CAT and GSH-Px or may react, in presence of O_2_^−^∙, with the ion Fe_2_^+^, via the Fenton and Haber-Weiss reaction, giving rise to reactive oxygen species (ROS). ROS lead to DNA, lipid and protein damage and apoptosis induction. In addition, anthracyclines can bind to NOS, causing an increase of RNS, particularly the ONOO^−^, highly harmful for cells. (**B**) Antimetabolites increase the levels of ROS/RNS mainly in cardiomyocytes and endothelial cells. In addition, antimetabolites can reduce the enzymatic activities of antioxidant enzymes. As a result, there is an imbalance between the production of ROS/RNS and the availability of antioxidants. This generates oxidative stress causing the oxidation of macromolecules, thus disturbing cellular functions. Antimetabolites, especially 5-FU, also lead to NOS dysregulation with reduced NO availability and increased RNS along with endothelin-1 upregulation and the activation of protein kinase C. These processes lead to endothelium-dependent and -independent vasoconstriction, and potentially to coronary spasm. (**C**) Finally, platinum-based antineoplastic agents induce oxidative stress by impairment of mitochondrial metabolism and nuclear activity, promoting apoptosis and cell death by BAX induction. In cardiomyocites, platinum-derivatives, primarily cisplatin, cause mitochondria dysfunction associated to increased caspase activity, ultimately leading to apoptosis. Abbreviations: CAT, catalase; GSH-Px, glutathione peroxidase; H_2_O_2_, hydrogen peroxide; NOS, nitrous oxide synthase; O_2_^−^∙, superoxide anion radical; ONOO^−^, peroxy-nitrite; RNS, reactive nitrogen species; ROS, reactive oxygen species; SOD, superoxide dismutase.

Thus far, the mechanism of anthracycline-related cardiotoxicity remains unclear, and is thought to be multifactorial and include ROS production. The most widely accepted hypothesis is that anthracyclines interfere with redox cycling and increase oxidative stress in cardiac cells where excessive ROS production causes mitochondrial dysfunction, calcium overload, DNA damage and lipid peroxidation of cardiac membranes (Figure 1A). Specifically, the quinone moiety of anthracyclines are susceptible to various cellular oxide reductases which catalyze univalent reduction to a semiquinone radical. In myocardial cells, this is predominantly achieved via an enzymatic pathway involving NADPH-cytochrome P450 reductase, a flavoprotein of the mitochondrial ETC. Successively, in the presence of molecular oxygen, the semiquinone auto-oxides generating the parent anthracycline and O_2_^−^ that are converted in oxygen by the semiquinone or undergo dismutation to form H_2_O_2_, either spontaneously or catalyzed by SOD. H_2_O_2_, via Fenton and Haber-Weiss reaction, reacts in turn with the transition metal ion Fe_2_^+^, toxic to cells as catalyst in the formation of free radicals. This forms the anthracycline-ferrous complex that is converted to anthracyclines-ferric complex by GSH-Px in the presence of flavoproteins. Lastly, generation of toxic hydroxyl radicals (OH^−^) triggers DNA and mitochondrial damage, lipid peroxidation, necrosis, apoptosis and finally leads to cell death. Vasquez et al. demonstrated that also peroxynitrite production and thus RNS generation plays a role in the cardiotoxicity [33]. Specifically, doxorubicin binds to the reductase domain of eNOS causing an increase in O_2_^−^ and a decrease in NO formation, bringing to endothelium-dependent and -independent vasoconstriction. From the combination of O_2_^−^, H_2_O_2_ and free iron, lipid peroxidation may be initiated [30,31,32]. 

### 3.2. Taxanes

Taxanes, belonging to a class of diterpenes with antineoplastic effects, are plant alkaloids. They are microtubule-stabilizing compounds that inhibit mitosis of cancer cells and interfere with a number of biochemical pathways to induce apoptosis and cancer cell death [34,35]. Among all the classes of antineoplastic agents, taxanes are one of the most widely used representing a cornerstone of chemotherapy. Taxanes are currently utilized in the management of multiple cancer types and in different setting of cancer treatment including adjuvant, neoadjuvant and metastatic setting. Paclitaxel, docetaxel and cabazitaxel are the most common taxanes utilized in oncology [35]. Taxanes cause cardiotoxicity in 2.3% to 8.0% of treated patients [36]. Typically, the risk of cardiotoxicity with taxanes is highest when they are used in combination or sequentially with anthracyclines [37]. This combinations is shown to induce high response rates in women with metastatic breast cancer, particularly when the two drugs are administered almost concomitantly [38,39]. Unfortunately, the clinical use of doxorubicin-paclitaxel combinations has been limited by an unexcepted high incidence of cardiotoxicity presumably because the taxanes act through an allosteric modulation of NADPH-dependent cytoplasmic reductases. As previously described, these enzymes play a major role in converting doxorubicin to its secondary alcohol metabolite doxorubicinol in the heart [37]. Doxorubicinol is an alcohol metabolite free radical that reduces oxygen to O_2_^−^ and its dis-mutated product H_2_O_2_. In the light of the low cardiac antioxidant mechanisms of hearth tissue as compared to other tissues, such ROS might play a role in inducing cardiotoxicity through mitochondrial dysfunction along with DNA damage, apoptosis, and lipid peroxidation. A less severe aggravation of cardiotoxicity is observed when taxanes are combined with epirubicin [40]. 

### 3.3. Antimetabolites

Antimetabolites such as fluoropyrimidines are cytotoxic drugs that can also induce cardiotoxicity. Fluoropyrimidines include capecitabine and 5-fluorouracil (5-FU). Both are currently approved for the treatment of several tumors including head and neck, breast, esophageal, gastric, pancreatic and colorectal cancer [41]. Capecitabine is converted into its active form, 5-FU, preferentially within tumors, by an enzyme expressed in both atherosclerotic plaques and cancer cells. Myocardial ischemia is the strongest risk factor for fluoropyrimidine related cardiotoxicity [42]. 5-FU, administered intravenously, has a short half-life, but active metabolites concentrate in cardiac and cancer cells, resulting in a prolonged exposure to the drug. The incidence of cardiotoxicity by 5-FU ranges from 1.0 to 18.0% [43], with a mortality rate between 2.0 and 13.0% [44]. In vitro and in vivo experiments demonstrated that fluoropyrimidines related cardiotoxicity is mediated by oxidative stress generation in cardiomyocytes as well as in endothelial cells [41,42,43,44,45,46] (Figure 1B). Specifically, ROS like O_2_^−^, are under normal physiological conditions cleared by antioxidant systems, such as SOD and GSH-Px. O_2_^−^ is dis-mutated to H_2_O_2_ in a process catalyzed by SOD, and H_2_O_2_ is then eliminated by catalase or GSH-Px. The activities of SOD and GSH-Px are impaired by 5-FU treatment demonstrating a reduced antioxidant capacity. If not eliminated by cellular antioxidant systems, O_2_^−^ can generate the highly reactive and toxic OH^−^ through the Haber–Weiss reaction catalyzed by iron. Increased ROS levels inside cells lead to oxidation of macromolecules including lipids, nucleic acids and proteins, thereby disturbing cellular functions. Moreover, 5-FU treatment causes eNOS dysregulation with inhibition of NO synthase and enhanced generation of RNS along with endothelin-1 upregulation and the activation of protein kinase C. This leads to endothelium-dependent and -independent vasoconstriction and eventually to coronary spasm [44,47]. 

### 3.4. Platinum-Based Antineoplastic Agents

Platinum-based antineoplastic drugs (informally called platins due to their coordination complexes of platinum) are also described as “alkylating-like” agents because their crosslinking in inhibiting DNA synthesis and repair in cancer cells. These chemotherapeutic agents are used to treat several cancer types. The most used platinum compounds in oncology are cisplatin, carboplatin and oxaliplatin but many others are under development [48]. 

Cisplatin, belonging to first generation of platinum compound, is the most frequently used [49] but dose-related nephrotoxicity and cardiotoxicity such as arrhythmias, angina pectoris, cardiac failures and vascular events limit its clinical use. These events may restrict the intensity and cumulative dose of cisplatin in some cases and increase the long-term prevalence of CVD in patients who have been received this type of chemotherapy [50,51]. The mechanisms underlying cisplatin-induced cardiotoxicity are not fully elucidated. However oxidative stress along with apoptosis, DNA damage and inflammation are most likely involved in the occurrence of cardiomyocytes injury (Figure 1C). Specifically, as confirmed in a variety of cytotoxicity models, cisplatin breaks the intracellular oxidative–antioxidant balance, leading to increased ROS production and cardiomyocyte apoptosis and necrosis through activation of Bax, a main regulator of Bcl-2 family [52]. When cisplatin accumulates in the mitochondrial matrix, it induces generation of ROS. This causes Bax activation and transport to the mitochondrial outer membrane where it leads to transition pores opening that alters mitochondrial permeability. This mechanism causes the release of the cytochrome *c* into the cytosol and consequent activation of pro-apoptotic factor release such as caspase 9 [53]. 

Carboplatin, a second-generation platinum compound, is also widely used for cancer treatment. Adverse events from carboplatin administration have a lower incidence as compared to cisplatin. As a result, it can be used at higher doses. The predominant dose-limiting toxicities of carboplatin are bone marrow suppression and ototoxicity [54] although some studies report cardiovascular adverse events such as atherosclerosis, hypertension and heart failure [55]. The mechanisms underlying carboplatin-mediated cardiovascular toxicity are still unclear. It is shown that ROS production and oxidative stress are also implicated [56]. 

Oxaliplatin, a third-generation platinum compound, is currently approved for the treatment of gastrointestinal cancer. It is less toxic as compared to cisplatin. While cumulative neuropathy is a common cause for treatment discontinuation, other toxicities are generally tolerable and managed by dose reductions and/or supportive treatment [57]. Nevertheless, acute, or chronic cardiotoxic adverse events have been also rarely observed. Patients with coronary artery spasm are at risk of ventricular arrhythmias and sudden cardiac death by oxaliplatin treatment [58]. The cardiomyocyte apoptosis by oxaliplatin is most likely caused by ROS, occurring via signal transducer and activator of transcription 1 (STAT1) and dual oxidase 2 (DUOX2) [59].

#### 3.4.1. Targeted Cancer Therapy

With the abundance of clinical research and a heavy focus on drug development, over the past decade, there has been a major change from non-specific cytotoxic drugs to molecularly targeted agents in cancer therapy. This change mostly involves the use of small-molecule tyrosine kinase inhibitors (TKIs), monoclonal antibodies (mAbs) and more recently immunotherapeutic modulators [60]. These agents inhibit the activity of upregulated cellular pathway components in malignant cells which drive tumor progression and/or immunoediting processes, leading to more effective and selective cancer therapy. 

The majority of the targeted therapeutic agents increases the oxidative stress burden to a level that is likely to overcome the reduction ability of cancer cells. In this way, targeted therapeutic agents display both a direct and oxidative stress mediated antitumor effect [59]. Despite their more selective anti-tumor effects as compared to classical chemotherapeutic agents, also use of targeted therapeutic agents may be limited by the development of both “on-target” and “off-target” adverse effects. One of the major “off-target” effect which emerges as a major safety concern is the reversible cardiotoxicity associated to hypertension, cardiac dysfunction and QT prolongation (Type 2 cardiotoxicity dysfunction) that is associated to an excessive ROS production [20,57,60]. As a result, early recognition and management of drug-related cardiotoxicity are extremely needed to reduce morbidity and mortality from this type of anti-cancer therapy. 

#### 3.4.2. TKIs

**Figure 2:**
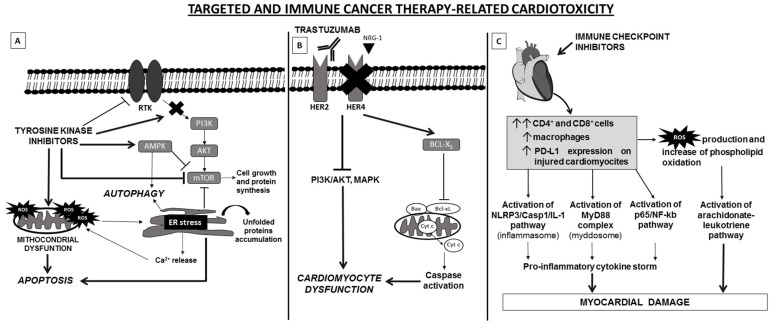
Schematic representation of molecular mechanisms of targeted and immune cancer therapy-related cardiotoxicity. (**A**) TKI administration induces cardiotoxicity via induction of ER stress and mitochondrial dysfunction, leading to unfolded protein accumulation, Ca2+ release, ROS generation, AMPK activation and mTOR inhibition. The interplay among these cellular pathways results in autophagy and apoptosis. (**B**) In heart, the cardioactive growth factor NRG-1 triggers HER-4/HER-2 heterodimerization to induce protective pathways in response to stress. Blockade of NRG-1/HER-4/HER-2 axis by trastuzumab results in the inhibition of MAPK and PI3K/AKT pathways which cause alterations of structure and functions of cardiomyocytes. In addition, blocking of HER2 is correlated with a change in the antiapoptotic/proapoptotic proteins ratio, due to an upregulation of BCL-XS (proapoptotic) and downregulation of BCL-XL (antiapoptotic), which leads to contractile dysfunction of cardiac cell. (**C**) ICI can induce cardiotoxicity by increasing in heart tissue infiltration of activated T lymphocytes (CD4+/CD8+ T cells) and macrophages and increased expression levels of PD-L1. This leads to pro-inflammatory and cardiotoxic effects and the activation of specific pathways, including NLRP3, Myd88, p65/NF-kb and arachidonate-leukotriene pathways. The latter probably is regulated by the high production of ROS and phospholipid oxidation due to accumulation of lymphocytes and macrophages. Abbreviations: AMPK, AMP-activated protein kinase; ER, endoplasmic reticulum; MAPK, mitogen-activated protein kinase; Myd88, myeloid differentiation primary response 88; mTOR, mammalian target of rapamycin; NLRP3, NLR Family Pyrin Domain Containing 3; NRG-1, neuregulin-1; PDL-1, Programmed death-ligand 1; ROS, Reactive oxygen species; RTK, receptor tyrosine kinase; TKIs, Tyrosine kinase inhibitors.

TKIs are low-weight molecules that act as antagonists of receptor tyrosine kinases (RTK) by (i) interfering with ATP-γ-phosphate for the ATP binding site within the kinase catalytic domain; (ii) preventing the auto-phosphorylation, dimerization and activation of intracellular pro-tumorigenic pathways; and (iii) inhibiting neo-angiogenesis and anti-apoptotic signals [61]. Many TKIs are designed to target vascular endothelial growth factor receptors (VEGFRs), platelet-derived growth factor receptors (PDGFRs), epidermal growth factor receptor (EGFR), fibroblast growth factor receptor (FGFR), HER-2, c-Kit, RET, Raf, Tie-2, ROS proto-oncogene 1 (ROS1) and c-Met; others to target the fusion protein Bcr-Abl, ALK and members of SRC or JAK tyrosine kinase family [62,63]. Currently, more than 50 TKIs are approved for the treatment of different types of cancer including non-small-cell lung cancer (NSCLC), gastrointestinal stromal tumor (GIST), hepatocellular carcinoma (HCC), renal cell carcinoma (RCC), breast, thyroid, ovarian, gastric and colon cancer as well as and hematological malignancies [64]. The degree of efficacy, selectivity and toxicity is variable among TKI treatments and depends by the inhibition of the specific kinase protein. Few TKIs specifically inhibit one kinase protein. Most of them inhibit multiple signaling pathway components based on their dose-dependent target affinity [64]. As a result, administration of TKIs is also associated to “off-target” effect. Cardiotoxicity from TKIs by cardiac mitochondrial damage is usually considered a drug’s “off-target” effect [62,64] (Figure 2A). Specifically, in cardiomyocytes the alteration of TK-related signaling by TKI administration causes endoplasmic reticulum (ER) stress and mitochondrial dysfunction. This in turn causes mitochondrial membrane permeabilization alteration, release of ROS into the cytoplasm, Ca^2+^ release, alterations of redox status and cell metabolism, and finally reprogramming of the phosphatidylinositol 3-kinase (PI3K), mammalian target of rapamycin (mTOR) and AMP-activated protein kinase (AMPK) signaling pathway. This leads to apoptosis induction and cell death [61]. 

Sunitinib was the first TKI approved for the treatment of advanced/metastatic RCC and currently there are also other clinical indications such as treatment of imatinib-resistant GISTs and pancreatic neuroendocrine carcinomas. Sunitinib acts by blocking VEGFRs, PDGFR-α, PDGFR-β and c-Kit. Its clinical use has been associated with several cardiovascular toxicities including hypertension, left ventricular (LV) dysfunction and heart failure. The mechanisms underlying cardiovascular toxicities from sunitinib are not fully elucidated [62,63,65]. Boutbir et al. aimed to enlarge their knowledge about the role of mitochondria dysfunction in sunitinib related cardiotoxicity in vitro and in vivo. They demonstrated that treatment with sunitinib dissipated the mitochondrial membrane potential and reduced enzyme complexes activities of the ETC along with an increased mitochondrial ROS accumulation. In addition, sunitinib caused caspase 3/7 activation, DNA fragmentation and increased H_2_O_2_ production. In mice, treatment with sunitinib, increased plasma concentrations of troponin I and creatine kinase myocardial band, indicating cardiomyocyte damage. The activity of enzyme complexes of the ETC was decreased, mitochondrial ROS were increased and cleavage of caspase 3 was increased, suggesting cardiomyocyte apoptosis induction. As a result, mitochondrial damage with ROS accumulation appears to be an important mechanism of cardiotoxicity associated with sunitinib, eventually leading to apoptotic cell death [65,66,67,68].

Besides sunitinib other TKIs such as pazopanib (anti-VEGFRs, PDGFR, FGFR and c-Kit), ponatinib (anti-Bcr-Abl), sorafenib and regorafenib (anti-VEGFRs, PDGFR, FGFR, c-Kit, RET, Raf, Tie2) are able to induce relevant mitochondrial dysfunction along with uncoupling components of ETC and ROS generation. Pazopanib is currently approved for treatment of RCC and non-adipocytic soft-tissue sarcoma. Its clinical efficacy is also limited by the related potential cardiovascular toxicity. The clinical use of pazopanib has been associated with the development of hypertension, QT interval prolongation and other cardiovascular events triggered by oxidative stress which in turn mediates apoptosis of cardiomyocytes [69]. Ponatinib is a third-generation TKI currently approved for the treatment of chronic myeloid leukemia (CML) in patients having gatekeeper mutation T315I and that are resistant to the first and second generation TKIs. Multiple unbiased screening has identified ponatinib as the most cardiotoxic clinically approved TKIs among the entire TKI family because of an “off-target” effect on cardiomyocyte pro-survival signaling pathway. This effect is mediated by inhibition of AKT and ERK signaling that leads to mitochondrial dysfunction and ROS production. This in turn causes DNA damage and apoptosis of cardiomyocytes [70,71]. Sorafenib is a multi-TKIs, currently approved for the treatment of RCC, HCC and differentiated thyroid cancer. The induction of oxidative stress and depletion of antioxidant status by sorafenib have been related to Ca^2+^ disturbances in hepatic tissue biopsy obtained from HCC patients. This effect is mediated by an increased ER stress as the levels of IRE1α, eukaryotic initiation factor 2α (eIF2α) and binding immunoglobulin protein (BiP)/glucose-regulated protein 78 (BiP/Grp78) were upregulated. Ca^2+^ disturbances leads to mitochondrial Ca^2+^ overload that induces lethal apoptotic events also in cardiomyocytes [61,65,66]. Regorafenib, currently approved for the treatment of colon cancer, HCC and GISTs directly uncouples oxidative phosphorylation and promotes Ca^2+^ accumulation and swelling of mitochondria, as demonstrated in human hepatic HepG2 cell line [71]. 

Imatinib, a PDGFR/Bcr-Abl/c-Kit TKI, currently approved for the treatment of acute lymphocytic leukemia (ALL), CML and GISTs has also been associated with cardiotoxicities by induction of apoptosis associated to ROS production and loss of the mitochondrial membrane potential [61]. 

Lastly, treatment with gefitinib and erlotinib (anti-EGFR TKIs) as well as with crizotinib (anti-ALK, ROS1 and c-Met) has been associated to cardiotoxicity. Gefitinib and erlotinib are currently approved for the treatment of NSCLC carrying EGFR alterations. Their related cardiotoxicity is associated to generation of NOX 4-induced oxidative stress [59]. Crizotinib, currently approved for the treatment of NSCLC carrying ALK translocation or ROS1 and c-Met mutations also exerts its functions via the induction of oxidative stress due to generation of ROS and activation of an apoptotic cascade that contributes to cardiomyocyte death [59,72].

#### 3.4.3. mAbs

mAb-based chemotherapy represents a relatively new and exciting class of treatment frequently utilized to improve new cancer treatment strategies for many hematological and solid tumors [73]. Since cancer cells share many similarities with the normal host cells, mAbs binding to specific tumor antigens on cell surface were engineered with the predicted advantage to display a high specificity to tumor antigens, directly targeting tumor cells and simultaneously promoting the induction of long-lasting anti-tumor immune responses [74]. Unfortunately, mAb-based therapy is also associated to deleterious effects on cardiomyocytes leading to heart failure, arrhythmias, myocardial ischemia, thromboembolism, hypertension and pericardial disease [75]. 

Trastuzumab was the first approved humanized mAb which blocks HER2 dimerization with other HER partners, evokes antibody-dependent cellular cytotoxicity and inhibits MAPK and PI3K/AKT pathways. Adding trastuzumab to standard chemotherapy, significantly improves response rates, progression free survival and overall survival of patients with metastatic HER2-positive breast cancer and gastric. Although trastuzumab has proven to be extremely effective, several studies have demonstrated that trastuzumab related cardiotoxicity represents a major limitation to its clinical use [76,77]. Retrospective analyses have shown that 27% of patients experienced cardiac dysfunction as a result of an adjuvant trastuzumab treatment [78]. Several studies have attempted to demonstrate the mechanism of trastuzumab induced cardiotoxicity (Figure 2B). Mohan et al. studied the effect of trastuzumab on autophagy in cardiomyocytes in a mouse model. The authors demonstrated that trastuzumab leads to phosphorylation of HER1-Y845/HER2-Y1248 and activation of ERK. This in turn results in upregulation of mTOR signaling pathway, inhibition of autophagy, accumulation of damaged mitochondria and increase concentration of ROS, nitrotyrosine and 4-hydroxynonenal, thereby triggering oxidative stress [76,79]. Kurokawa et al. replicated the trastuzumab-induced cardiotoxicity using human stem cells in vitro with aim to better define the molecular events. The authors demonstrated that cardiomyocytes originating from induced pluripotent stem cells (iPS-CMs) are able to replicate the cardiotoxicity of trastuzumab-inhibited HER signaling. Although this discovery have acquired important implications for cancer treatment in humans, the researchers were only able to detect cardiotoxicity when iPS-CMs were also treated with doxorubicin [80]. 

Pertuzumab, a new recombinant humanized mAb, inhibits HER2 dimerization with other HER family receptors. It is currently approved for the treatment of breast cancer in combination with trastuzumab to enhance anti-HER2 efficacy. Pertuzumab significantly enhances the cardiotoxicity induced by trastuzumab. Blocking HER2 receptor induces mitochondrial dysfunction and ROS overload production that mediate cardiomyocyte cell death [81]. With the same mechanism rituximab, an anti-CD20 mAb currently approved for the treatment of CLL and non-Hodgkin lymphoma (NHL), and bevacizumab, an anti-VEGF mAb currently approved for the treatment of multiple cancer types including breast, colorectal, cervical, NSCLC, ovarian and RCC have been reported to be cardiotoxic due to an oxidative stress induction. The most lethal cardiac issues with them are encountered during an infusion reaction for the development of hypotension, hypoxia, acute myocardial infarction, arrythmias and cardiogenic shock [59].

#### 3.4.4. Immune Checkpoint Inhibitor Based Immunotherapy

Over the past few years, cancer immunotherapies have revolutionized the clinical management of a wide spectrum of solid and hematological malignancies. The forefront of immunotherapy is represented by immune checkpoint inhibitors (ICIs). ICI inhibit immune checkpoint molecule such as cytotoxic-T-lymphocyte-associated antigen 4 (CTLA-4), programmed cell death 1 (PD-1) and its ligand PD-L1. These molecules are involved in regulating the host immune response to both cancer cells and altered cells. ICIs such as mAbs targeting CTLA-4 (ipilimumab), PD-1 (nivolumab, pembrolizumab) and PD-L1 (atezolizumab, avelumab, durvalumab) restore an efficient anti-tumor immune response. However immune checkpoint molecules also play a central role in the maintenance of immune self-tolerance. Therefore, blocking these molecules by mAbs can result in the development of immune-related adverse events. This type of toxicity has a high incidence, but fortunately in most cases it is reversible and not severe [82]. Every normal tissue may be involved in this type of toxicity. Isolated cases of fulminant myocarditis and other cardiovascular disorders (pericarditis, vasculitis and atrioventricular blocks) have been reported [83]. Little is known about the mechanisms underlying ICI-related cardiotoxicity and no ROS-mediated effects has been described yet. However, a reasonable amount of data exists to point out that an oxidative milieu has an enormous impact on tumor cells, tumor-infiltrating lymphocytes and other immune cells (and their interactions) (Figure 2C). It is plausible that these agents have direct or indirect ROS-dependent mechanisms arising from interactions between PD-1 antibodies and ROS generation [59]. Both ROS levels and redox status have been reported to have a potential role as a prognostic and predictive biomarker to immunotherapy. Therefore, studies addressing these issues are eagerly awaited. 

## 4. Molecules to Fight the Onset and Progression of CVD

In reason of the crucial role of oxidative stress in CVD, pharmacological and non-pharmacological therapeutic approaches have been proposed to prevent an increase of pro-oxidant species and/or to enhance the capacity of the endogenous antioxidant systems [84,85]. Several studies have evaluated the potential therapeutic effects of antioxidant molecules to fight the onset and progression of CVD including that induced by anti-cancer agents [86,87]. Although various studies both in vitro and in vivo showed cardioprotective effects from antioxidative molecules, clinical trials have failed to demonstrate a clinical benefit from this type of therapeutic approach and no specific antioxidative treatment has been recommended, so far [86,87,88,89,90]. Here, we will summarize the molecules which have been tested in order to reduce the oxidative stress and to improve CVD outcomes (Table 1). We will describe firstly the potential role of vitamins and other nutraceuticals as polyphenols and astaxanthin. Then, we will outline the emerging evidence of antioxidative properties of some drugs already utilized in clinical practice as NO donors, antihypertensives, antidiabetics and statins. Finally, we will speculate about the use of novel molecular pathway inhibitors which potentially revert the oxidative stress in CVD.

### 4.1. Vitamins and Nutraceuticals

#### 4.1.1. Vitamins

Several studies showed a potential therapeutic benefit of vitamins in decreasing oxidative stress both in cellular and mouse models [86,91]. Specifically, vitamin C and folic acid have been shown to display a cardioprotective effect, preventing NOS uncoupling and restoring endothelial dysfunction [91,92,93,94]. Moreover, hyperhomocysteinemia plays an important role in oxidative stress-mediated endothelial dysfunction [86,139,140] ]. It is well known that homocysteine levels are mitigated by administration of folic acid, vitamin B12 and B6 [86]. Hagar et al. demonstrated that vitamin B12 and folic acid administration on isoprenaline-induced myocardial infarction restores heart rate, blood pressure and ST segment elevation in an experimental hyperhomocysteinemic rat model [95]. Farhangi et al. demonstrated the potential benefit of vitamin D administration on cardiac stress and inflammation in obese rats [96]. Similarly, antioxidative effects were demonstrated in mouse models in which administration of vitamin E promoted restoration of cardiac function and attenuation of atherogenic apo B-48-dependent hyperlipidemia [97,98,99]. Based on these results, many clinical studies have attempted to demonstrate the potential antioxidant effect of vitamins [88,141]. However, none of tested vitamins significantly demonstrated its potential clinical benefit in CVD [86,87,88,89,90]. Possible reasons of this failure include the difference of oxidative stress levels in patients that were not assessed and stratified for their oxidative stress status [88,142]. The modality and duration of vitamin administration as well as the potential interaction of vitamins with other potential antioxidant drugs may have also influenced the obtained results [88,143]. As discussed later, many drugs exerting a potent antioxidative effects such as statin, antihypertensive and anti-diabetic agents were often administered in these trials to patients because of their comorbidity [88,91,131,144]. In conclusion, although vitamin supplementation has the potential to overcome the onset and progression of CVD, there is not sufficient clinical evidence to translate these results in the clinical practice and novel clinical studies are urgent needed. 

#### 4.1.2. Polyphenols

Polyphenols are common natural, semi-synthetic and synthetic molecules, composed of multiples phenol units derived from fruits, vegetables, tea, coffee, cocoa, mushrooms, beverages and traditional medical herbs [145,146]. The classification of polyphenols mainly includes flavonoids (60%), phenolic acids (30%) and others polyphenols (as stilbenes and lignans) [145,146,147]. Previous studies showed that polyphenols can scavenge O_2_^−^ and peroxynitrite [147]. Furthermore, they exert other antioxidative effects by regulating oxidative stress-mediated enzyme activities [147,148] and chelation of the transition metals involved in radical-forming processes [147,148,149]. Cells respond to polyphenol exposition by triggering a series of redox-dependent reactions and inducing a modification of redox status of the cells [147,150,151]. For these reasons, polyphenols have been investigated as potential co-adjuvants in malignant, CVD, metabolic and neurodegenerative diseases [147,152,153].. Just like for other types of supplemental nutrients also for the polyphenols are yielded contrasting in vivo results [86,87,147,154] and their supplementation is not yet recommended in clinical practice.

#### 4.1.3. Astaxanthin

Astaxanthin, a carotenoid of the xanthophyll group, has important applications in the nutraceutical, cosmetics, food and feed industries [103,155,156]. Chemical structure of astaxanthin includes two oxygenated groups, hydroxyl and carbonyl, in each a ionone ring displays some unique properties such as more effective antioxidative activity and polar configuration as compared to other carotenoids [103,104,155,156,157,158]. Moreover, astaxanthin exerts some anti-inflammatory effects and modulates the lipid and glucose metabolisms [103,104,105,106,107]. In reason of these multiple pleiotropic effects, several studies have demonstrated that astaxanthin can delay the onset and progression of various CVD such as atherosclerosis, hypertension and dyslipidemia [103,104,105,106,107,159,160,161,162]. Considering the extensive evidence reported in the literature about its benefit–risk profile, astaxanthin has been approved as a nutraceutical by the United States Food and Drug Administration [163].

### 4.2. Anti-Oxidative Properties of Common Drugs

#### 4.2.1. NO Donors

As we have previously mentioned, NO depletion promotes endothelial dysfunction that is crucial in CVD pathophysiology of coronary artery atherosclerosis [164]. Administration of molecules able to restore the redox balance through exogenous administration of NO donors as organic nitrates has been investigated as a potential strategy to fight the onset and progression of CVD [91]. These molecules such as the isosorbide-5-mononitrate are currently used to decrease the excessive coronary artery constriction since they induce NO and/or NO derived molecule releasing by endothelial cells [165]. Since atherosclerosis is closely linked to endothelial dysfunction, some researchers have proposed that nitrates by acting as NO donors on endothelium might also decrease the onset and progression of atherosclerosis [91,166,167]. On the other hand, in the few past years, several lines of evidence demonstrated that organic nitrates cause endothelium dysfunction by increasing oxidative stress mediated by (i) uncoupling of the mitochondrial respiratory chain and decreasing of ATP production; (ii) activation of ROS-forming enzymes and (iii) direct reaction of the nitrate-derived NO with vascular O_2_^−^ to form highly reactive intermediate peroxynitrite (ONOO^−^) [91,168,169,170,171,172]. Based on these contrasting results the interest for NO donors as a potential strategy to fight oxidative stress is hampered and novel and robust evidence are needed. 

#### 4.2.2. Anti-Hypertensives

Antihypertensive classes including angiotensin converting enzyme (ACE) inhibitors, angiotensin receptor (AT) blockers, beta receptor blockers and calcium channel blockers have demonstrated so many clinical benefits that are not fully ascribed by their primary mechanisms of action [91]. ACE inhibitors improve the prognosis of patients with multiple cardiovascular risk factors, acute myocardial infarction, chronic congestive heart failure, diabetic nephropathy and other vascular diseases [118,119,120,121,122]. These clinical benefits have been also associated to their antioxidative properties. Indeed, various cardiovascular protective effects mediated by an antioxidative property of ACE inhibitors have been demonstrated [91,144]. The antioxidative effect of ACE inhibitors is most likely mediated by the blockage of renin-angiotensin axis. Angiotensin II induces vasoconstriction but also promotes atherosclerosis through monocyte-macrophage recruitment into vessel wall, smooth muscle cell mitogenesis and extracellular matrix storage [91,123,124,125,126]. Moreover, angiotensin II is one of the most important triggers for the NOX activity. As we previously mentioned, this enzyme is expressed on the surface of all vascular endothelial cells and represents an important source of O_2_^−^ [91,164,173]. As a result, renin-angiotensin axis activation is constantly involved in oxidative stress through ROS production [91,164,173,174]. In addition, ACE, working as an endothelial kininase II, leads to the degradation of bradykinin. The latter via B_2_ receptor induces the release of vasodilator and antioxidative molecules such as NO, endothelium-derived hyperpolarizing factor and prostacyclin [91,108,109,110]. Thus, ACE inhibitors exert an important antioxidative activity by incrementing bradykinin levels and inhibiting NOXs. 

AT blockers act by blocking the angiotensin receptor I. Similarly to ACE inhibitors also AT blockers display a pleiotropic antioxidative effects that derives from renin-angiotensin axis blockage [100,111,112,113]. However, in reason of the different mechanism of action from ACE inhibitors, AT blockers cannot exert the bradykinin-mediated antioxidative effects. Nevertheless, several studies including clinical trials have demonstrated the clinical benefits of AT blockers in fighting the onset and progression of CVD [101,102,114,115,116].

Besides inhibiting adrenaline/noradrenaline beta adrenergic receptors, beta receptor blockers also exert antioxidative properties [175,176,177,178,179]. Carvedilol, celiprolol, metoprolol, nebivolol, propranolol and their antioxidative mechanisms are the most extensively investigated [144]. Each beta receptor blocker presents its different antioxidative profile in terms of efficacy and mechanism [175,176,177,178,179,180,181]. Carvedilol and metoprolol, by acting as a free radical scavenger, reduce lipid peroxidation in patient affected by symptomatic stable heart failure [182]. Celiprolol reduces O_2_^−^ generation in patients with essential hypertension and improves myocardial remodeling [144,183,184,185,186]. Nebivolol reduces antioxidative stress in essential hypertension patients by increasing NO formation [117,180,187]. Propranolol inhibits oxidative stress by reducing lipid peroxidation of sarcolemma [135]. Lastly, some lines of evidence have shown that beta receptor blockers such as celiprolol can reduce blood pressure levels and mitigate hypoxia-induced left ventricle remodeling by restoring eNOS expression through stimulation of PI3K/AKT signaling pathway [127,136,144].

Calcium channel blockers have also been investigated for their potential antioxidative properties in a minor extent as compared to the above described antihypertensive drugs [127,128,129]. Amlodipine decreases oxidative stress by reducing malondialdehyde and increasing erythrocyte sodium-potassium adenosine triphosphatase and SOD levels in essential hypertensive patients [127].

#### 4.2.3. Anti-Diabetic Agents

Dipeptidyl peptidase-4 (DDP-4) inhibitors, glucagon-like peptide-1 (GLP-1) analogues and sodium-glucose cotransporter 2 (SGLT2) inhibitors are the most innovative anti-diabetic agents. Besides their anti-diabetic primary effect, several lines of evidence demonstrated that these drugs reduce the risk of hospitalization for heart failure [130,131,188,189]. The cardiovascular protective effects of these anti-diabetic agents are most likely mediated by anti-oxidative and anti-inflammatory properties [131,190,191,192,193,194,195,196]. For instance, linagliptin, a DDP-4 inhibitor, and liraglutide (GLP-1 analogue) reduce oxidative burst in whole blood, augment the infiltration of white blood cells through the vascular wall and reduce the expression of NADPH oxidase [131,132,133]. Steven et al. demonstrated that these antioxidative mechanisms can be mediated by GLP-1 induced cAMP and protein kinase A elevation [131,133]. 

In addition SGLT2 inhibitors such as ipragliflozin display many antioxidative mediated cardiovascular protective properties beyond their primary anti-diabetic effect [197,198]. Empagliflozin treatment reduces oxidative stress in the aorta and blood of diabetic rats [131,134]. Even more. inflammation and glucotoxicity are epigenetically prevented by SGLT2 inhibitors by NOS2 and IFNɣ reduction in vivo [131,134]. Although these novel classes of anti-diabetic drugs have such potential antioxidant properties no clinical evidence are currently available to recommend their clinical use. 

#### 4.2.4. Statins

Statins are a lipid lowering drugs able to improve the prognosis of patients affected by coronary or peripheral arterial disease, heart failure, hypercholesterolemia and several other conditions by inhibiting 3-hydroxy-3-methylglutaryl-coenzyme A reductase [199,200,201,202,203,204]. Furthermore statins exert additional pleiotropic properties such as improvement of endothelial function, stabilization of atherosclerotic plaques, reduction of both oxidative stress and inflammation, and decrease of thrombogenic responses [205,206,207,208,209]. These antioxidative effects are mediated by the increase of eNOS activity and the reduction of both asymmetrical dimethylarginine levels and NADPH oxidase function [91,137,138]. Due to these multiple pleiotropic activities on the protective role in CVD, further investigations are needed to established the real impact of their antioxidative effects in CVD [91]. 

## 5. Novel Potential Strategy to Revert Antioxidative Stress in CVD

### 5.1. Mitochondrial-Targeted Antioxidative Based Therapy

As described above, ROS production is an important factor in CVD onset and progression. Classically, mitochondria are considered one of the most important actors engaged in ROS production [210]. Indeed, various strategies targeting mitochondria, such as small molecules have been tested in preclinical studies in order to fight the onset and the progression of CVD [86,87,211]. A mitochondria-targeted SOD mimetic molecule, called mito-TEMPO, decreases the mitochondrial O_2_^−^ and H_2_O_2_ production both in cellular and mouse models [86,212,213]. Specifically, mito-TEMPO reduces angiotensin II induced hypertension, the mitochondrial O_2_^−^ and 3-nitrotyrosine activity as well as serum glucose levels and diastolic dysfunction in high-fat diet mice [86,213,214]. Furthermore, mito-TEMPO administration prevents cardiomyocytes hypertrophy in diabetic mouse hearts by decreasing mitochondrial ROS production [86,212]. 

On the other hand, contrasting results have been shown from mitoquinone (MitoQ) use, another mitochondrial-targeted oxidative drug. Although some evidence showed that MitoQ mitigates redox-induced cardio-toxicities and improves cardiac mitochondrial network integrity [86,215,216], other studies in cancer cell models, demonstrated that MitoQ decreases mitochondrial DNA integrity and induces apoptosis by enhancing ROS production and mitochondrial membrane depolarization [86,217,218]. Thus, although promising further studies are needed to demonstrate the efficacy of this drug in humans. 

### 5.2. mTOR Signaling Pathway Inhibition

mTOR is a serine/threonine kinase protein which plays a crucial role in many cellular processes such as cell growth, metabolism, proliferation, survival, transcription, translation, apoptosis. motility and autophagy [219,220]. In reason of several mTOR functions, several lines of evidence showed that mTOR pathway dysregulation is involved in many diseases including CVD [221,222]. However, the relationship between mTOR pathway and oxidative stress in CVD is still to be elucidated since this pathway is involved both in oxidative stress mitigation and production [221,223]. Moreover, although potential regulator effect on oxidative stress through mTOR signaling down-regulation has been shown in animal models of experimental diabetic conditions, clinical studies are still disappointing [223]. Several molecules have demonstrated to exert many antioxidative cardioprotective effects by regulating the mTOR signaling pathway [221,223]. These molecules included (i) inhibitors of mTOR such as rapamycin and its derivates [224,225,226,227,228], and ii) molecules that in different ways modulate mTOR pathway such as metformin [221,223,229], resveratrol [221,230,231,232,233], curcumin [221,234,235] and sirtuin 1 [223,236]. 

Although, mTOR signaling pathway inhibition seems to be an interesting novel potential strategy to fight the onset and the progression of oxidative stress in CVD, likewise for mitochondrial-targeted anti-oxidative based therapies, more studies, especially in humans, are needed to validate this type of strategy. 

## 6. Conclusions

Oxidative stress is certainly one of the most important factors in CVD as well as in cardiotoxicity pathogenesis. Despite a large mass of studies, all the mechanisms underlying the role of stress oxidative in both CVD and cardiotoxicity remains unclear. Moreover, no one of the currently therapeutic strategies can effectively revert oxidative stress in clinical practice, so far. Many questions remain open and several investigations are still needed.

## Figures and Tables

**Table 1:** Molecules and Drugs to overcome oxidative stress inducing CVD.

Nutraceutical/Class of Drug	Main Mechanism of Action	Additional Antioxidative Effects	References
Vitamins and nutraceuticals			
B6	Metabolic Coenzyme in several cellular processes	Reduction of homocysteine levels	[86]
Acid folic (B9)	Metabolic Coenzyme in several cellular processes	(i) Prevention of NOS uncoupling and restoration of endothelial dysfunction (ii) Reduction of homocysteine levels (iii) Heart rate and blood pressure restoration	[86,91,92,93,94,95]
B12	Metabolic Coenzyme in several cellular processes	(i) Reduction of homocysteine levels (ii) Heart rate and blood pressure restoration	[86,91,92,93,94,95]
C	Antioxidative	Prevention of NOS uncoupling and restoration of endothelial dysfunction	[91,92,93,94]
D	Regulation of calcium metabolism	Improvement of cardiac stress and inflammation in obese rats	[96]
E	Antioxidative	Restoration of cardiac function and attenuation of atherogenic apo B-48-dependent hyperlipidemia	[97,98,99]
Polyphenols	Antioxidative	(i) O_2_^−^ and peroxynitrite scavenger (ii) Inducers of redox dependent reactions	[100,101,102]
Astaxanthin	Antioxidative	(i) Anti-inflammatory effects (ii) Regulation of lipid and glucose metabolisms	[103,104,105,106,107]
Anti-hypertensives			
Angiotensin converting enzyme (ACE) inhibitors	ACE inhibition	(i) Reduction of monocyte-macrophage recruitment into vessel wall, smooth muscle cells mitogenesis and extracellular matrix storage (ii) Reduction of ACE mediated ROS production (iii) Increase of bradykinin levels	[91,108,109,110]
Angiotensin receptor (AT) blockers	AT blockage	Pleiotropic anti-oxidative effects derived from renin-angiotensin axis blockage without bradykinin-mediated antioxidative effects	[100,101,102,111,112,113,114,115,116]
Beta adrenergic receptor blockers	Beta adrenergic receptor blockage	(i) Free radical scavenger function (ii) Decrease of superoxide anions generation (iii) Restoring eNOS expression	[117,118,119,120,121,122,123,124,125,126,127,128,129,130]
Anti-diabetic agents			
Dipeptidyl peptidase-4 (DDP-4) inhibitors	DDP-4 inhibition	(i) Decrease of oxidative burst in whole blood (ii) Decrease of the expression of NADPH oxidase (iii) Elevation cAMP and protein kinase A	[131,132,133]
Glucagon-like peptide-1 (GLP-1) analogues	GLP-1 functions	(i) Decrease of oxidative burst in whole blood (ii) Decrease of the expression of NADPH oxidase (iii) Elevation cAMP and protein kinase A	[131,132,133]
Sodium-glucose cotransporter 2 (SGLT2) inhibitors	SGLT2 blockage	NOS2 and IFNɣ reduction	[131,134]
Others			
NO donors	NO and/or NO derived molecule releasing by endothelial cells	NO and/or NO derived molecule releasing by endothelial cells restoring REDOX balance	[92,131,135,136]
Statins	3-hydroxy-3-methylglutaryl-coenzyme A reductase inhibition	(i) Increase of eNOS activity (ii) Decrease of both asymmetrical dimethylarginine levels and NADPH oxidase function	[91,137,138]

Abbreviations: ACE: angiotensin converting enzyme, AT: angiotensin receptor, CVD: cardiovascular diseases, DDP-4: Dipeptidyl peptidase-4, eNOS: endothelial NO synthase, GLP-1: glucagon-like peptide-1, NO: nitric oxide, ROS: radical oxygen species, SGLT2: sodium-glucose cotransporter 2.

## Abbreviations

ACE	angiotensin converting enzyme
ALL	acute lymphocytic leukemia
AMPK	AMP-activated protein kinase
AT	angiotensin receptor
ATP	adenosine triphosphate
BiP	binding immunoglobulin protein
CML	chronic myeloid leukemia
CTLA-4	cytotoxic-T-lymphocyte-associated antigen 4
CVD	cardiovascular diseases
DDP-4	Dipeptidyl peptidase-4
DUOX2	dual oxidase 2
EGFR	epidermal growth factor receptor
eIF2α	eukaryotic initiation factor 2α
eNOS	endothelial NO synthase
ER	endoplasmic reticulum
ETC	electron transfer chain
FGFR	fibroblast growth factor receptor
GIST	gastrointestinal stromal tumor
GLP-1	glucagon-like peptide-1
Grp78	glucose-regulated protein 78
GSH-Px	glutathione peroxidase
HCC	hepatocellular carcinoma
hPSC	human pluripotent stem cell
H_2_O_2_	hydrogen peroxide
ICI	immune checkpoint inhibitors
iPS-CMs	induced pluripotent stem cells
LV	left ventricular
mAbs	monoclonal antibodies
MitoQ	mitoquinone
mTOR	mammalian target of rapamycin
NHL	non-Hodgkin lymphoma
NO	nitric oxide
NOXs	NADH/NADPH oxidases
NSCLC	non-small-cell-lung-cancer
ONOO^−^	peroxynitrite
O_2_^−^	superoxide
PDGFR	platelet-derived growth factor receptor
PD-1	programmed cell death 1
PI3K	phosphatidylinositol 3-kinase
RCC	renal cell carcinoma
RNS	reactive nitrogen species
ROS	radical oxygen species
ROS1	ROS proto-oncogene 1
RTK	receptor tyrosine kinases
SGLT2	sodium-glucose cotransporter 2
SOD	superoxide dismutase
STAT1	signal transducer and activator of transcription 1
TKIs	tyrosine kinase inhibitors
VEGFR	vascular endothelial growth factor receptors
5-FU	5-fluorouracil
OH^−^	hydroxyl radicals

## References

[1] Valko M., Leibfritz D., Moncol J., Cronin M.T.D., Mazur M., Telser J. (2007). Free Radicals and Antioxidants in Normal Physiological Functions and Human Disease. Int. J. Biochem. Cell Biol..

[2] Nordberg J., Arnér E.S. (2001). Reactive Oxygen Species, Antioxidants, and the Mammalian Thioredoxin System. Free Radic. Biol. Med..

[3] Ristow M., Schmeisser K. (2014). Mitohormesis: Promoting Health and Lifespan by Increased Levels of Reactive Oxygen Species (ROS). Dose Response.

[4] Cao S.S., Kaufman R.J. (2014). Endoplasmic Reticulum Stress and Oxidative Stress in Cell Fate Decision and Human Disease. Antioxid. Redox Signal..

[5] Conti V., Corbi G., Simeon V., Russomanno G., Manzo V., Ferrara N., Filippelli A. (2015). Aging-Related Changes in Oxidative Stress Response of Human Endothelial Cells. Aging Clin. Exp. Res..

[6] Sack M.N., Fyhrquist F.Y., Saijonmaa O.J., Fuster V., Kovacic J.C. (2017). Basic Biology of Oxidative Stress and the Cardiovascular System: Part 1 of a 3-Part Series. J. Am. Coll. Cardiol..

[7] Ciancarelli I., di Massimo C., de Amicis D., Carolei A., Ciancarelli M.G.T. (2012). Evidence of Redox Unbalance in Post-Acute Ischemic Stroke Patients. Curr. Neurovasc. Res..

[8] Conti V., Forte M., Corbi G., Russomanno G., Formisano L., Landolfi A., Izzo V., Filippelli A., Vecchione C., Carrizzo A. (2017). Sirtuins: Possible Clinical Implications in Cardio and Cerebrovascular Diseases. Curr. Drug Targets.

[9] Brand M.D. (2010). The Sites and Topology of Mitochondrial Superoxide Production. Exp. Gerontol..

[10] Kujoth G.C., Hiona A., Pugh T.D., Someya S., Panzer K., Wohlgemuth S.E., Hofer T., Seo A.Y., Sullivan R., Jobling W.A. (2005). Mitochondrial DNA Mutations, Oxidative Stress, and Apoptosis in Mammalian Aging. Science.

[11] Otín C.L., Blasco M.A., Partridge L., Serrano M., Kroemer G. (2013). The Hallmarks of Aging. Cell.

[12] Minamino T., Komuro I. (2008). Vascular Aging: Insights from Studies on Cellular Senescence, Stem Cell Aging, and Progeroid Syndromes. Nat. Clin. Pract. Cardiovasc. Med..

[13] Glancy B., Balaban R.S. (2012). Role of Mitochondrial Ca2+ in the Regulation of Cellular Energetics. Biochemistry.

[14] Molkentin J.D., Dorn G.W. (2001). Cytoplasmic Signaling Pathways That Regulate Cardiac Hypertrophy. Annu. Rev. Physiol..

[15] Radi E., Formichi P., Battisti C., Federico A. (2014). Apoptosis and Oxidative Stress in Neurodegenerative Diseases. J. Alzheimers Dis..

[16] Tublin J.M., Adelstein J.M., del Monte F., Combs C.K., Wold L.E. (2019). Getting to the Heart of Alzheimer Disease. Circ. Res..

[17] Cai H., Harrison D.G. (2000). Endothelial Dysfunction in Cardiovascular Diseases: The Role of Oxidant Stress. Circ. Res..

[18] Harrison D.G. (1997). Endothelial Function and Oxidant Stress. Clin. Cardiol..

[19] Zhang P.-Y., Xu X., Li X.-C. (2014). Cardiovascular Diseases: Oxidative Damage and Antioxidant Protection. Eur. Rev. Med. Pharmacol. Sci..

[20] Perez I.E., Alam S.T., Hernandez G.A., Sancassani R. (2019). Cancer Therapy-Related Cardiac Dysfunction: An Overview for the Clinician. Clin. Med. Insights Cardiol..

[21] Castaldo S.A., Freitas J.R., Conchinha N.V., Madureira P.A. (2016). The Tumorigenic Roles of the Cellular REDOX Regulatory Systems. Oxid. Med. Cell. Longev..

[22] Angsutararux P., Luanpitpong S., Issaragrisil S. (2015). Chemotherapy-Induced Cardiotoxicity: Overview of the Roles of Oxidative Stress. Oxid. Med. Cell. Longev..

[23] Herman E., Eldridge S. (2019). Spontaneously Occurring Cardiovascular Lesions in Commonly Used Laboratory Animals. Cardiooncology.

[24] Liu X., Wang X., Zhang X., Xie Y., Chen R., Chen H. (2012). C57BL/6 Mice Are More Appropriate than BALB/C Mice in Inducing Dilated Cardiomyopathy with Short-Term Doxorubicin Treatment. Acta Cardiol. Sin..

[25] Qi W., Boliang W., Xiaoxi T., Guoqiang F., Jianbo X., Gang W. (2020). Cardamonin Protects against Doxorubicin-Induced Cardiotoxicity in Mice by Restraining Oxidative Stress and Inflammation Associated with Nrf2 Signaling. Biomed. Pharmacother..

[26] Chakraborti S., Pramanick A., Saha S., Roy S.S., Chaudhuri A.R., das M., Ghosh S., Stewart A., Maity B. (2017). Atypical G Protein Β5 Promotes Cardiac Oxidative Stress, Apoptosis, and Fibrotic Remodeling in Response to Multiple Cancer Chemotherapeutics. Cancer Res..

[27] Cappetta D., de Angelis A., Sapio L., Prezioso L., Illiano M., Quaini F., Rossi F., Berrino L., Naviglio S., Urbanek K. (2017). Oxidative Stress and Cellular Response to Doxorubicin: A Common Factor in the Complex Milieu of Anthracycline Cardiotoxicity. Oxid. Med. Cell. Longev..

[28] Schwach V., Slaats R.H., Passier R. (2020). Human Pluripotent Stem Cell-Derived Cardiomyocytes for Assessment of Anticancer Drug-Induced Cardiotoxicity. Front. Cardiovasc. Med..

[29] Lipshultz S.E., Herman E.H. (2018). Anthracycline Cardiotoxicity: The Importance of Horizontally Integrating Pre-Clinical and Clinical Research. Cardiovasc. Res..

[30] Marinello J., Delcuratolo M., Capranico G. (2018). Anthracyclines as Topoisomerase II Poisons: From Early Studies to New Perspectives. Int. J. Mol. Sci..

[31] McGowan J.V., Chung R., Maulik A., Piotrowska I., Walker J.M., Yellon D.M. (2017). Anthracycline Chemotherapy and Cardiotoxicity. Cardiovasc. Drugs Ther..

[32] Henriksen P.A. (2018). Anthracycline Cardiotoxicity: An Update on Mechanisms, Monitoring and Prevention. Heart.

[33] Vivar J.V., Martasek P., Hogg N., Masters B.S., Pritchard K.A., Kalyanaraman B. (1997). Endothelial Nitric Oxide Synthase-Dependent Superoxide Generation from Adriamycin. Biochemistry.

[34] Gjyrezi A., Xie F., Voznesensky O., Khanna P., Calagua C., Bai Y., Kung J., Wu J., Corey E., Montgomery B. (2020). Taxane Resistance in Prostate Cancer Is Mediated by Decreased Drug-Target Engagement. J. Clin. Investig..

[35] Fitzpatrick J.M., de Wit R. (2014). Taxane Mechanisms of Action: Potential Implications for Treatment Sequencing in Metastatic Castration-Resistant Prostate Cancer. Eur. Urol..

[36] Ng R., Better N., Green M.D. (2006). Anticancer Agents and Cardiotoxicity. Semin. Oncol..

[37] Perotti A., Cresta S., Grasselli G., Capri G., Minotti G., Gianni L. (2003). Cardiotoxic Effects of Anthracycline-Taxane Combinations. Expert Opin. Drug Saf..

[38] Putt M., Hahn V.S., Januzzi J.L., Sawaya H., Sebag I.A., Plana J.C., Picard M.H., Carver J.R., Halpern E.F., Kuter I. (2015). Longitudinal Changes in Multiple Biomarkers Are Associated with Cardiotoxicity in Breast Cancer Patients Treated with Doxorubicin, Taxanes, and Trastuzumab. Clin. Chem..

[39] Bines J., Earl H., Buzaid A.C., Saad E.D. (2014). Anthracyclines and Taxanes in the Neo/Adjuvant Treatment of Breast Cancer: Does the Sequence Matter?. Ann. Oncol..

[40] Salvatorelli E., Menna P., Gianni L., Minotti G. (2007). Defective Taxane Stimulation of Epirubicinol Formation in the Human Heart: Insight into the Cardiac Tolerability of Epirubicin-Taxane Chemotherapies. J. Pharmacol. Exp. Ther..

[41] Deac A.-L., Burz C.C., Bocşe H.F., Bocşan I.C., Buzoianu A.-D. (2020). A Review on the Importance of Genotyping and Phenotyping in Fluoropyrimidine Treatment. Med. Pharm. Rep..

[42] Layoun M.E., Wickramasinghe C.D., Peralta M.V., Yang E.H. (2016). Fluoropyrimidine-Induced Cardiotoxicity: Manifestations, Mechanisms, and Management. Curr. Oncol. Rep..

[43] Yuan C., Parekh H., Allegra C., George T.J., Starr J.S. (2019). 5-FU Induced Cardiotoxicity: Case Series and Review of the Literature. Cardiooncology.

[44] Polk A., Vistisen K., Nilsen M.V., Nielsen D.L. (2014). A Systematic Review of the Pathophysiology of 5-Fluorouracil-Induced Cardiotoxicity. BMC Pharmacol. Toxicol..

[45] Lamberti M., Porto S., Marra M., Zappavigna S., Grimaldi A., Feola D., Pesce D., Naviglio S., Spina A., Sannolo N. (2012). 5-Fluorouracil Induces Apoptosis in Rat Cardiocytes through Intracellular Oxidative Stress. J. Exp. Clin. Cancer Res..

[46] Millart H., Brabant L., Lorenzato M., Lamiable D., Albert O., Choisy H. (1992). The Effects of 5-Fluorouracil on Contractility and Oxygen Uptake of the Isolated Perfused Rat Heart. Anticancer Res..

[47] Durak I., Karaayvaz M., Kavutcu M., Cimen M.Y., Kaçmaz M., Büyükkoçak S., Oztürk H.S. (2000). Reduced Antioxidant Defense Capacity in Myocardial Tissue from Guinea Pigs Treated with 5-Fluorouracil. J. Toxicol. Environ. Health A.

[48] Dilruba S., Kalayda G.V. (2016). Platinum-Based Drugs: Past, Present and Future. Cancer Chemother. Pharmacol..

[49] Makovec T. (2019). Cisplatin and beyond: Molecular Mechanisms of Action and Drug Resistance Development in Cancer Chemotherapy. Radiol. Oncol..

[50] Hu Y., Sun B., Zhao B., Mei D., Gu Q., Tian Z. (2018). Cisplatin-Induced Cardiotoxicity with Midrange Ejection Fraction: A Case Report and Review of the Literature. Medicine (Baltimore).

[51] Demkow U., Emmel A.S. (2013). Cardiotoxicity of Cisplatin-Based Chemotherapy in Advanced Non-Small Cell Lung Cancer Patients. Respir. Physiol. Neurobiol..

[52] Marullo R., Werner E., Degtyareva N., Moore B., Altavilla G., Ramalingam S.S., Doetsch P.W. (2013). Cisplatin Induces a Mitochondrial-ROS Response That Contributes to Cytotoxicity Depending on Mitochondrial Redox Status and Bioenergetic Functions. PLoS ONE.

[53] Qian P., Yan L.-J., Li Y.-Q., Yang H.-T., Duan H.-Y., Wu J.-T., Fan X.-W., Wang S.-L. (2018). Cyanidin Ameliorates Cisplatin-Induced Cardiotoxicity via Inhibition of ROS-Mediated Apoptosis. Exp. Ther. Med..

[54] Ho G.Y., Woodward N., Coward J.I.G. (2016). Cisplatin versus Carboplatin: Comparative Review of Therapeutic Management in Solid Malignancies. Crit. Rev. Oncol. Hematol..

[55] Cheng C.-F., Juan S.-H., Chen J.-J., Chao Y.-C., Chen H.-H., Lian W.-S., Lu C.-Y., Chang C.-I., Chiu T.-H., Lin H. (2008). Pravastatin Attenuates Carboplatin-Induced Cardiotoxicity via Inhibition of Oxidative Stress Associated Apoptosis. Apoptosis.

[56] He P.-J., Ge R.-F., Mao W.-J., Chung P.-S., Ahn J.-C., Wu H.-T. (2018). Oxidative Stress Induced by Carboplatin Promotes Apoptosis and Inhibits Migration of HN-3 Cells. Oncol. Lett..

[57] Alcindor T., Beauger N. (2011). Oxaliplatin: A Review in the Era of Molecularly Targeted Therapy. Curr. Oncol..

[58] Polyzos A., Tsavaris N., Gogas H., Souglakos J., Vambakas L., Vardakas N., Polyzos K., Tsigris C., Mantas D., Papachristodoulou A. (2009). Clinical Features of Hypersensitivity Reactions to Oxaliplatin: A 10-Year Experience. Oncology.

[59] Teppo H.-R., Soini Y., Karihtala P. (2017). Reactive Oxygen Species-Mediated Mechanisms of Action of Targeted Cancer Therapy. Oxid. Med. Cell. Longev..

[60] Xie Y.-H., Chen Y.-X., Fang J.-Y. (2020). Comprehensive Review of Targeted Therapy for Colorectal Cancer. Signal Transduct. Target. Ther..

[61] Hernández M.A.R., de la Cruz-Ojeda P., Grueso M.J.L., Villarán E.N., Aguilar R.R., Vega B.C., Negrete M., Gallego P., Ochoa Á.V., Victor V.M. (2020). Integrated Molecular Signaling Involving Mitochondrial Dysfunction and Alteration of Cell Metabolism Induced by Tyrosine Kinase Inhibitors in Cancer. Redox Biol..

[62] Yamaoka T., Kusumoto S., Ando K., Ohba M., Ohmori T. (2018). Receptor Tyrosine Kinase-Targeted Cancer Therapy. Int. J. Mol. Sci..

[63] Pottier C., Fresnais M., Gilon M., Jérusalem G., Longuespée R., Sounni N.E. (2020). Tyrosine Kinase Inhibitors in Cancer: Breakthrough and Challenges of Targeted Therapy. Cancers.

[64] Broekman F., Giovannetti E., Peters G.J. (2011). Tyrosine Kinase Inhibitors: Multi-Targeted or Single-Targeted?. World J. Clin. Oncol..

[65] Chaar M., Kamta J., Oudhia S.A. (2018). Mechanisms, Monitoring, and Management of Tyrosine Kinase Inhibitors-Associated Cardiovascular Toxicities. OncoTargets Ther..

[66] Chu T.F., Rupnick M.A., Kerkela R., Dallabrida S.M., Zurakowski D., Nguyen L., Woulfe K., Pravda E., Cassiola F., Desai J. (2007). Cardiotoxicity Associated with Tyrosine Kinase Inhibitor Sunitinib. Lancet.

[67] Narayan V., Keefe S., Haas N., Wang L., Puzanov I., Putt M., Catino A., Fang J., Agarwal N., Hyman D. (2017). Prospective Evaluation of Sunitinib-Induced Cardiotoxicity in Patients with Metastatic Renal Cell Carcinoma. Clin. Cancer Res..

[68] Bouitbir J., Alshaikhali A., Panajatovic M.V., Abegg V.F., Paech F., Krähenbühl S. (2019). Mitochondrial Oxidative Stress Plays a Critical Role in the Cardiotoxicity of Sunitinib: Running Title: Sunitinib and Oxidative Stress in Hearts. Toxicology.

[69] Justice C.N., Derbala M.H., Baich T.M., Kempton A.N., Guo A.S., Ho T.H., Smith S.A. (2018). The Impact of Pazopanib on the Cardiovascular System. J. Cardiovasc. Pharmacol. Ther..

[70] Singh A.P., Umbarkar P., Tousif S., Lal H. (2020). Cardiotoxicity of the BCR-ABL1 Tyrosine Kinase Inhibitors: Emphasis on Ponatinib. Int. J. Cardiol..

[71] Paech F., Mingard C., Grünig D., Abegg V.F., Bouitbir J., Krähenbühl S. (2018). Mechanisms of Mitochondrial Toxicity of the Kinase Inhibitors Ponatinib, Regorafenib and Sorafenib in Human Hepatic HepG2 Cells. Toxicology.

[72] Du X., Shao Y., Qin H.-F., Tai Y.-H., Gao H.-J. (2018). ALK-Rearrangement in Non-Small-Cell Lung Cancer (NSCLC). Thorac. Cancer.

[73] Scott A.M., Allison J.P., Wolchok J.D. (2012). Monoclonal Antibodies in Cancer Therapy. Cancer Immun..

[74] Coulson A., Levy A., Williams M.G. (2014). Monoclonal Antibodies in Cancer Therapy: Mechanisms, Successes and Limitations. West Indian Med. J..

[75] Hansel T.T., Kropshofer H., Singer T., Mitchell J.A., George A.J.T. (2010). The Safety and Side Effects of Monoclonal Antibodies. Nat. Rev. Drug Discov..

[76] Genuino A.J., Chaikledkaew U., The D.O., Reungwetwattana T., Thakkinstian A. (2019). Adjuvant Trastuzumab Regimen for HER2-Positive Early-Stage Breast Cancer: A Systematic Review and Meta-Analysis. Expert Rev. Clin. Pharmacol..

[77] Balduzzi S., Mantarro S., Guarneri V., Tagliabue L., Pistotti V., Moja L., D’Amico R. (2014). Trastuzumab-Containing Regimens for Metastatic Breast Cancer. Cochrane Database Syst. Rev..

[78] Guarneri V., Lenihan D.J., Valero V., Durand J.-B., Broglio K., Hess K.R., Michaud L.B., Angulo A.M.G., Hortobagyi G.N., Esteva F.J. (2006). Long-Term Cardiac Tolerability of Trastuzumab in Metastatic Breast Cancer: The M.D. Anderson Cancer Center Experience. J. Clin. Oncol..

[79] Mohan N., Jiang J., Wu W.J. (2017). Implications of Autophagy and Oxidative Stress in Trastuzumab-Mediated Cardiac Toxicities. Austin Pharmacol. Pharm..

[80] Kurokawa Y.K., Shang M.R., Yin R.T., George S.C. (2018). Modeling Trastuzumab-Related Cardiotoxicity in Vitro Using Human Stem Cell-Derived Cardiomyocytes. Toxicol. Lett..

[81] Sendur M.A.N., Aksoy S., Altundag K. (2015). Pertuzumab-Induced Cardiotoxicity: Safety Compared with Trastuzumab. Future Oncol..

[82] Zhang Y., Zhang Z. (2020). The History and Advances in Cancer Immunotherapy: Understanding the Characteristics of Tumor-Infiltrating Immune Cells and Their Therapeutic Implications. Cell. Mol. Immunol..

[83] Varricchi G., Galdiero M.R., Marone G., Criscuolo G., Triassi M., Bonaduce D., Marone G., Tocchetti C.G. (2017). Cardiotoxicity of Immune Checkpoint Inhibitors. ESMO Open.

[84] Sosnowska B., Penson P., Banach M. (2017). The Role of Nutraceuticals in the Prevention of Cardiovascular Disease. Cardiovasc. Diagn. Ther..

[85] Russomanno G., Corbi G., Manzo V., Ferrara N., Rengo G., Puca A.A., Latte S., Carrizzo A., Calabrese M.C., Andriantsitohaina R. (2017). The Anti-Ageing Molecule Sirt1 Mediates Beneficial Effects of Cardiac Rehabilitation. Immun. Ageing.

[86] Deruy E.D., Peugnet V., Turkieh A., Pinet F. (2020). Oxidative Stress in Cardiovascular Diseases. Antioxidants.

[87] Senoner T., Dichtl W. (2019). Oxidative Stress in Cardiovascular Diseases: Still a Therapeutic Target?. Nutrients.

[88] Poznyak A.V., Grechko A.V., Orekhova V.A., Chegodaev Y.S., Wu W.-K., Orekhov A.N. (2020). Oxidative Stress and Antioxidants in Atherosclerosis Development and Treatment. Biology.

[89] Pignatelli P., Menichelli D., Pastori D., Violi F. (2018). Oxidative Stress and Cardiovascular Disease: New Insights. Kardiol. Pol..

[90] Tousoulis D., Briasoulis A., Papageorgiou N., Tsioufis C., Tsiamis E., Toutouzas K., Stefanadis C. (2011). Oxidative Stress and Endothelial Function: Therapeutic Interventions. Recent Pat. Cardiovasc. Drug Discov..

[91] Gori T., Münzel T. (2011). Oxidative Stress and Endothelial Dysfunction: Therapeutic Implications. Ann. Med..

[92] Moat S.J., Clarke Z.L., Madhavan A.K., Lewis M.J., Lang D. (2006). Folic Acid Reverses Endothelial Dysfunction Induced by Inhibition of Tetrahydrobiopterin Biosynthesis. Eur. J. Pharmacol..

[93] Hyndman M.E., Verma S., Rosenfeld R.J., Anderson T.J., Parsons H.G. (2002). Interaction of 5-Methyltetrahydrofolate and Tetrahydrobiopterin on Endothelial Function. Am. J. Physiol. Heart Circ. Physiol..

[94] Stanhewicz A.E., Kenney W.L. (2017). Role of Folic Acid in Nitric Oxide Bioavailability and Vascular Endothelial Function. Nutr. Rev..

[95] Hagar H.H. (2002). FOLIC ACID AND VITAMIN B12 SUPPLEMENTATION ATTENUATES ISOPRENALINE-INDUCED MYOCARDIAL INFARCTION IN EXPERIMENTAL HYPERHOMOCYSTEINEMIC RATS. Pharmacol. Res..

[96] Farhangi M.A., Nameni G., Hajiluian G., Mesgari-Abbasi M. (2017). Cardiac Tissue Oxidative Stress and Inflammation after Vitamin D Administrations in High Fat- Diet Induced Obese Rats. BMC Cardiovasc. Disord..

[97] Contreras-Duarte S., Chen P., Andía M., Uribe S., Irarrázaval P., Kopp S., Kern S., Marsche G., Busso D., Wadsack C. (2018). Attenuation of Atherogenic Apo B-48-Dependent Hyperlipidemia and High Density Lipoprotein Remodeling Induced by Vitamin C and E Combination and Their Beneficial Effect on Lethal Ischemic Heart Disease in Mice. Biol. Res..

[98] Wallert M., Ziegler M., Wang X., Maluenda A., Xu X., Yap M.L., Witt R., Giles C., Kluge S., Hortmann M. (2019). α-Tocopherol Preserves Cardiac Function by Reducing Oxidative Stress and Inflammation in Ischemia/Reperfusion Injury. Redox Biol..

[99] Wang X., Dong W., Yuan B., Yang Y., Yang D., Lin X., Chen C., Zhang W. (2016). Vitamin E Confers Cytoprotective Effects on Cardiomyocytes under Conditions of Heat Stress by Increasing the Expression of Metallothionein. Int. J. Mol. Med..

[100] Yao E.-H., Fukuda N., Matsumoto T., Kobayashi N., Katakawa M., Yamamoto C., Tsunemi A., Suzuki R., Ueno T., Matsumoto K. (2007). Losartan Improves the Impaired Function of Endothelial Progenitor Cells in Hypertension via an Antioxidant Effect. Hypertens. Res..

[101] Al Khalaf M.M., Thalib L., Doi S.A.R. (2009). Cardiovascular Outcomes in High-Risk Patients without Heart Failure Treated with ARBs: A Systematic Review and Meta-Analysis. Am. J. Cardiovasc. Drugs.

[102] Miura S., Saku K. (2014). Recent Progress in the Treatment of Cardiovascular Disease Using Olmesartan. Clin. Exp. Hypertens.

[103] Pereira C.P.M., Souza A.C.R., Vasconcelos A.R., Prado P.S., Name J.J. (2020). Antioxidant and Anti-inflammatory Mechanisms of Action of Astaxanthin in Cardiovascular Diseases (Review). Int. J. Mol. Med..

[104] Fassett R.G., Coombes J.S. (2009). Astaxanthin, Oxidative Stress, Inflammation and Cardiovascular Disease. Future Cardiol..

[105] Iwamoto T., Hosoda K., Hirano R., Kurata H., Matsumoto A., Miki W., Kamiyama M., Itakura H., Yamamoto S., Kondo K. (2000). Inhibition of Low-Density Lipoprotein Oxidation by Astaxanthin. J. Atheroscler. Thromb..

[106] Kishimoto Y., Yoshida H., Kondo K. (2016). Potential Anti-Atherosclerotic Properties of Astaxanthin. Mar. Drugs.

[107] Mashhadi N.S., Zakerkish M., Mohammadiasl J., Zarei M., Mohammadshahi M., Haghighizadeh M.H. (2018). Astaxanthin Improves Glucose Metabolism and Reduces Blood Pressure in Patients with Type 2 Diabetes Mellitus. Asia Pac. J. Clin. Nutr..

[108] Mombouli J.V., Vanhoutte P.M. (1994). Kinins and the Vascular Actions of Converting Enzyme Inhibitors. Curr. Opin. Nephrol. Hypertens.

[109] Wirth K.J., Linz W., Wiemer G., Schölkens B.A. (1997). Kinins and Cardioprotection. Pharmacol. Res..

[110] Tschöpe C., Gohlke P., Zhu Y.Z., Linz W., Schölkens B., Unger T. (1997). Antihypertensive and Cardioprotective Effects after Angiotensin-Converting Enzyme Inhibition: Role of Kinins. J. Card. Fail..

[111] Honjo T., Yamaoka-Tojo M., Inoue N. (2011). Pleiotropic Effects of ARB in Vascular Metabolism—Focusing on Atherosclerosis-Based Cardiovascular Disease. Curr. Vasc. Pharmacol..

[112] Yu Y., Fukuda N., Yao E.-H., Matsumoto T., Kobayashi N., Suzuki R., Tahira Y., Ueno T., Matsumoto K. (2008). Effects of an ARB on Endothelial Progenitor Cell Function and Cardiovascular Oxidation in Hypertension. Am. J. Hypertens..

[113] Suzuki R., Fukuda N., Katakawa M., Tsunemi A., Tahira Y., Matsumoto T., Ueno T., Soma M. (2014). Effects of an Angiotensin II Receptor Blocker on the Impaired Function of Endothelial Progenitor Cells in Patients with Essential Hypertension. Am. J. Hypertens..

[114] Chrysant S.G. (2008). Angiotensin II Receptor Blockers in the Treatment of the Cardiovascular Disease Continuum. Clin. Ther..

[115] Sabbah Z.A., Mansoor A., Kaul U. (2013). Angiotensin Receptor Blockers—Advantages of the New Sartans. J. Assoc. Physicians India.

[116] Akhrass P.R., McFarlane S.I. (2011). Telmisartan and Cardioprotection. Vasc. Health Risk Manag..

[117] Wang Y., Zhang F., Liu Y., Yin S., Pang X., Li Z., Wei Z. (2017). Nebivolol Alleviates Aortic Remodeling through ENOS Upregulation and Inhibition of Oxidative Stress in L-NAME-Induced Hypertensive Rats. Clin. Exp. Hypertens..

[118] Gums J.G. (1992). Use of ACE Inhibitors in the Treatment of Cardiovascular Disease. Am. Pharm..

[119] Borghi C., Cosentino E., De Sanctis D. (2005). Angiotensin-converting enzyme inhibition and cardiovascular prevention: More than twenty years of clinical success. Ital. Heart J. Suppl..

[120] Song J.C., White C.M. (2002). Clinical Pharmacokinetics and Selective Pharmacodynamics of New Angiotensin Converting Enzyme Inhibitors: An Update. Clin. Pharm..

[121] Hoyer J., Schulte K.L., Lenz T. (1993). Clinical Pharmacokinetics of Angiotensin Converting Enzyme (ACE) Inhibitors in Renal Failure. Clin. Pharm..

[122] Beckwith C., Munger M.A. (1993). Effect of Angiotensin-Converting Enzyme Inhibitors on Ventricular Remodeling and Survival Following Myocardial Infarction. Ann. Pharm..

[123] Tummala P.E., Chen X.L., Sundell C.L., Laursen J.B., Hammes C.P., Alexander R.W., Harrison D.G., Medford R.M. (1999). Angiotensin II Induces Vascular Cell Adhesion Molecule-1 Expression in Rat Vasculature: A Potential Link between the Renin-Angiotensin System and Atherosclerosis. Circulation.

[124] Pueyo M.E., Gonzalez W., Nicoletti A., Savoie F., Arnal J.F., Michel J.B. (2000). Angiotensin II Stimulates Endothelial Vascular Cell Adhesion Molecule-1 via Nuclear Factor-KappaB Activation Induced by Intracellular Oxidative Stress. Arter. Thromb. Vasc. Biol..

[125] Chen X.L., Tummala P.E., Olbrych M.T., Alexander R.W., Medford R.M. (1998). Angiotensin II Induces Monocyte Chemoattractant Protein-1 Gene Expression in Rat Vascular Smooth Muscle Cells. Circ. Res..

[126] Touyz R.M., He G., El Mabrouk M., Diep Q., Mardigyan V., Schiffrin E.L. (2001). Differential Activation of Extracellular Signal-Regulated Protein Kinase 1/2 and P38 Mitogen Activated-Protein Kinase by AT1 Receptors in Vascular Smooth Muscle Cells from Wistar-Kyoto Rats and Spontaneously Hypertensive Rats. J. Hypertens..

[127] Mahajan A.S., Babbar R., Kansal N., Agarwal S.K., Ray P.C. (2007). Antihypertensive and Antioxidant Action of Amlodipine and Vitamin C in Patients of Essential Hypertension. J. Clin. Biochem. Nutr..

[128] Umemoto S., Tanaka M., Kawahara S., Kubo M., Umeji K., Hashimoto R., Matsuzaki M. (2004). Calcium Antagonist Reduces Oxidative Stress by Upregulating Cu/Zn Superoxide Dismutase in Stroke-Prone Spontaneously Hypertensive Rats. Hypertens. Res..

[129] Kouoh F., Gressier B., Dine T., Luyckx M., Brunet C., Ballester L., Cazin J.C. (2002). Antioxidant Effects and Anti-Elastase Activity of the Calcium Antagonist Nicardipine on Activated Human and Rabbit Neutrophils--a Potential Antiatherosclerotic Property of Calcium Antagonists?. Cardiovasc. Drugs Ther..

[130] Fadini G.P., Avogaro A., Degli Esposti L., Russo P., Saragoni S., Buda S., Rosano G., Pecorelli S., Pani L. (2015). OsMed Health-DB Network Risk of Hospitalization for Heart Failure in Patients with Type 2 Diabetes Newly Treated with DPP-4 Inhibitors or Other Oral Glucose-Lowering Medications: A Retrospective Registry Study on 127,555 Patients from the Nationwide OsMed Health-DB Database. Eur. Heart J..

[131] Steven S., Frenis K., Oelze M., Kalinovic S., Kuntic M., Bayo Jimenez M.T., Vujacic-Mirski K., Helmstädter J., Kröller-Schön S., Münzel T. (2019). Vascular Inflammation and Oxidative Stress: Major Triggers for Cardiovascular Disease. Oxid. Med. Cell. Longev..

[132] Kröller-Schön S., Knorr M., Hausding M., Oelze M., Schuff A., Schell R., Sudowe S., Scholz A., Daub S., Karbach S. (2012). Glucose-Independent Improvement of Vascular Dysfunction in Experimental Sepsis by Dipeptidyl-Peptidase 4 Inhibition. Cardiovasc. Res..

[133] Steven S., Jurk K., Kopp M., Kröller-Schön S., Mikhed Y., Schwierczek K., Roohani S., Kashani F., Oelze M., Klein T. (2017). Glucagon-like Peptide-1 Receptor Signalling Reduces Microvascular Thrombosis, Nitro-Oxidative Stress and Platelet Activation in Endotoxaemic Mice. Br. J. Pharmacol..

[134] Steven S., Oelze M., Hanf A., Kröller-Schön S., Kashani F., Roohani S., Welschof P., Kopp M., Gödtel-Armbrust U., Xia N. (2017). The SGLT2 Inhibitor Empagliflozin Improves the Primary Diabetic Complications in ZDF Rats. Redox Biol..

[135] Mak I.T., Weglicki W.B. (1988). Protection by Beta-Blocking Agents against Free Radical-Mediated Sarcolemmal Lipid Peroxidation. Circ. Res..

[136] Kobayashi N., Mita S., Yoshida K., Honda T., Kobayashi T., Hara K., Nakano S., Tsubokou Y., Matsuoka H. (2003). Celiprolol Activates ENOS through the PI3K-Akt Pathway and Inhibits VCAM-1 Via NF-KappaB Induced by Oxidative Stress. Hypertension.

[137] Lu T.-M., Ding Y.-A., Leu H.-B., Yin W.-H., Sheu W.H.-H., Chu K.-M. (2004). Effect of Rosuvastatin on Plasma Levels of Asymmetric Dimethylarginine in Patients with Hypercholesterolemia. Am. J. Cardiol..

[138] Wassmann S., Laufs U., Müller K., Konkol C., Ahlbory K., Bäumer A.T., Linz W., Böhm M., Nickenig G. (2002). Cellular Antioxidant Effects of Atorvastatin in Vitro and in Vivo. Arterioscler. Thromb. Vasc. Biol..

[139] Stamler J.S., Osborne J.A., Jaraki O., Rabbani L.E., Mullins M., Singel D., Loscalzo J. (1993). Adverse Vascular Effects of Homocysteine Are Modulated by Endothelium-Derived Relaxing Factor and Related Oxides of Nitrogen. J. Clin. Invest..

[140] McCully K.S. (1992). Homocystinuria, Arteriosclerosis, Methylmalonic Aciduria, and Methyltransferase Deficiency: A Key Case Revisited. Nutr. Rev..

[141] Libby P., Everett B.M. (2019). Novel Antiatherosclerotic Therapies. Arterioscler. Thromb. Vasc. Biol..

[142] Martin-Ventura J.L., Rodrigues-Diez R., Martinez-Lopez D., Salaices M., Blanco-Colio L.M., Briones A.M. (2017). Oxidative Stress in Human Atherothrombosis: Sources, Markers and Therapeutic Targets. Int. J. Mol. Sci..

[143] Violi F., Loffredo L., Carnevale R., Pignatelli P., Pastori D. (2017). Atherothrombosis and Oxidative Stress: Mechanisms and Management in Elderly. Antioxid. Redox Signal..

[144] Sorriento D., De Luca N., Trimarco B., Iaccarino G. (2018). The Antioxidant Therapy: New Insights in the Treatment of Hypertension. Front. Physiol..

[145] Neveu V., Perez-Jiménez J., Vos F., Crespy V., du Chaffaut L., Mennen L., Knox C., Eisner R., Cruz J., Wishart D. (2010). Phenol-Explorer: An Online Comprehensive Database on Polyphenol Contents in Foods. Database (Oxford).

[146] Manach C., Scalbert A., Morand C., Rémésy C., Jiménez L. (2004). Polyphenols: Food Sources and Bioavailability. Am. J. Clin. Nutr..

[147] Cheng Y.-C., Sheen J.-M., Hu W.L., Hung Y.-C. (2017). Polyphenols and Oxidative Stress in Atherosclerosis-Related Ischemic Heart Disease and Stroke. Oxid. Med. Cell. Longev..

[148] Ramos S. (2008). Cancer Chemoprevention and Chemotherapy: Dietary Polyphenols and Signalling Pathways. Mol. Nutr. Food Res..

[149] Thomas R., Kim M.H. (2005). Epigallocatechin Gallate Inhibits HIF-1alpha Degradation in Prostate Cancer Cells. Biochem. Biophys. Res. Commun..

[150] Moskaug J.Ø., Carlsen H., Myhrstad M.C.W., Blomhoff R. (2005). Polyphenols and Glutathione Synthesis Regulation. Am. J. Clin. Nutr..

[151] Halliwell B., Rafter J., Jenner A. (2005). Health Promotion by Flavonoids, Tocopherols, Tocotrienols, and Other Phenols: Direct or Indirect Effects? Antioxidant or Not?. Am. J. Clin. Nutr..

[152] Middleton E., Kandaswami C., Theoharides T.C. (2000). The Effects of Plant Flavonoids on Mammalian Cells: Implications for Inflammation, Heart Disease, and Cancer. Pharmacol. Rev..

[153] Chuang C.-C., McIntosh M.K. (2011). Potential Mechanisms by Which Polyphenol-Rich Grapes Prevent Obesity-Mediated Inflammation and Metabolic Diseases. Annu. Rev. Nutr..

[154] Banez M.J., Geluz M.I., Chandra A., Hamdan T., Biswas O.S., Bryan N.S., Von Schwarz E.R. (2020). A Systemic Review on the Antioxidant and Anti-Inflammatory Effects of Resveratrol, Curcumin, and Dietary Nitric Oxide Supplementation on Human Cardiovascular Health. Nutr. Res..

[155] Guerin M., Huntley M.E., Olaizola M. (2003). Haematococcus Astaxanthin: Applications for Human Health and Nutrition. Trends Biotechnol..

[156] Hussein G., Sankawa U., Goto H., Matsumoto K., Watanabe H. (2006). Astaxanthin, a Carotenoid with Potential in Human Health and Nutrition. J. Nat. Prod..

[157] Yuan J.-P., Peng J., Yin K., Wang J.-H. (2011). Potential Health-Promoting Effects of Astaxanthin: A High-Value Carotenoid Mostly from Microalgae. Mol. Nutr. Food Res..

[158] Zhang L., Wang H. (2015). Multiple Mechanisms of Anti-Cancer Effects Exerted by Astaxanthin. Mar. Drugs.

[159] Choi H.D., Youn Y.K., Shin W.G. (2011). Positive Effects of Astaxanthin on Lipid Profiles and Oxidative Stress in Overweight Subjects. Plant Foods Hum. Nutr..

[160] Nakagawa K., Kiko T., Miyazawa T., Carpentero Burdeos G., Kimura F., Satoh A., Miyazawa T. (2011). Antioxidant Effect of Astaxanthin on Phospholipid Peroxidation in Human Erythrocytes. Br. J. Nutr..

[161] Karppi J., Rissanen T.H., Nyyssönen K., Kaikkonen J., Olsson A.G., Voutilainen S., Salonen J.T. (2007). Effects of Astaxanthin Supplementation on Lipid Peroxidation. Int. J. Vitam. Nutr. Res..

[162] Yoshida H., Yanai H., Ito K., Tomono Y., Koikeda T., Tsukahara H., Tada N. (2010). Administration of Natural Astaxanthin Increases Serum HDL-Cholesterol and Adiponectin in Subjects with Mild Hyperlipidemia. Atherosclerosis.

[163] Lorenz R.T., Cysewski G.R. (2000). Commercial Potential for Haematococcus Microalgae as a Natural Source of Astaxanthin. Trends Biotechnol..

[164] Münzel T., Sinning C., Post F., Warnholtz A., Schulz E. (2008). Pathophysiology, Diagnosis and Prognostic Implications of Endothelial Dysfunction. Ann. Med..

[165] Kleschyov A.L., Oelze M., Daiber A., Huang Y., Mollnau H., Schulz E., Sydow K., Fichtlscherer B., Mülsch A., Münzel T. (2003). Does Nitric Oxide Mediate the Vasodilator Activity of Nitroglycerin?. Circ. Res..

[166] Kojda G., Stein D., Kottenberg E., Schnaith E.M., Noack E. (1995). In Vivo Effects of Pentaerythrityl-Tetranitrate and Isosorbide-5-Mononitrate on the Development of Atherosclerosis and Endothelial Dysfunction in Cholesterol-Fed Rabbits. J. Cardiovasc. Pharmacol..

[167] Kojda G., Noack E. (1995). Effects of Pentaerythrityl-Tetranitrate and Isosorbide-5-Mononitrate in Experimental Atherosclerosis. Agents Actions Suppl..

[168] Wenzel P., Mollnau H., Oelze M., Schulz E., Wickramanayake J.M.D., Müller J., Schuhmacher S., Hortmann M., Baldus S., Gori T. (2008). First Evidence for a Crosstalk between Mitochondrial and NADPH Oxidase-Derived Reactive Oxygen Species in Nitroglycerin-Triggered Vascular Dysfunction. Antioxid. Redox Signal..

[169] Gori T., Burstein J.M., Ahmed S., Miner S.E., Al-Hesayen A., Kelly S., Parker J.D. (2001). Folic Acid Prevents Nitroglycerin-Induced Nitric Oxide Synthase Dysfunction and Nitrate Tolerance: A Human in Vivo Study. Circulation.

[170] Gori T., Mak S.S., Kelly S., Parker J.D. (2001). Evidence Supporting Abnormalities in Nitric Oxide Synthase Function Induced by Nitroglycerin in Humans. J. Am. Coll. Cardiol..

[171] Münzel T., Daiber A., Mülsch A. (2005). Explaining the Phenomenon of Nitrate Tolerance. Circ. Res..

[172] Gori T., Parker J.D. (2008). Nitrate-Induced Toxicity and Preconditioning: A Rationale for Reconsidering the Use of These Drugs. J. Am. Coll. Cardiol..

[173] Warnholtz A., Nickenig G., Schulz E., Macharzina R., Bräsen J.H., Skatchkov M., Heitzer T., Stasch J.P., Griendling K.K., Harrison D.G. (1999). Increased NADH-Oxidase-Mediated Superoxide Production in the Early Stages of Atherosclerosis: Evidence for Involvement of the Renin-Angiotensin System. Circulation.

[174] Guzik T.J., West N.E., Black E., McDonald D., Ratnatunga C., Pillai R., Channon K.M. (2000). Vascular Superoxide Production by NAD(P)H Oxidase: Association with Endothelial Dysfunction and Clinical Risk Factors. Circ. Res..

[175] Dandona P., Ghanim H., Brooks D.P. (2007). Antioxidant Activity of Carvedilol in Cardiovascular Disease. J. Hypertens.

[176] Feuerstein G.Z., Ruffolo R.R. (1996). Carvedilol, a Novel Vasodilating Beta-Blocker with the Potential for Cardiovascular Organ Protection. Eur. Heart J..

[177] Nakamura K., Murakami M., Miura D., Yunoki K., Enko K., Tanaka M., Saito Y., Nishii N., Miyoshi T., Yoshida M. (2011). Beta-Blockers and Oxidative Stress in Patients with Heart Failure. Pharmaceuticals.

[178] Wang R., Miura T., Harada N., Kametani R., Shibuya M., Fukagawa Y., Kawamura S., Ikeda Y., Hara M., Matsuzaki M. (2006). Pleiotropic Effects of the Beta-Adrenoceptor Blocker Carvedilol on Calcium Regulation during Oxidative Stress-Induced Apoptosis in Cardiomyocytes. J. Pharmacol. Exp. Ther..

[179] Ni L., Zhou C., Duan Q., Lv J., Fu X., Xia Y., Wang D.W. (2011). β-AR Blockers Suppresses ER Stress in Cardiac Hypertrophy and Heart Failure. PLoS ONE.

[180] Fratta Pasini A., Garbin U., Nava M.C., Stranieri C., Davoli A., Sawamura T., Lo Cascio V., Cominacini L. (2005). Nebivolol Decreases Oxidative Stress in Essential Hypertensive Patients and Increases Nitric Oxide by Reducing Its Oxidative Inactivation. J. Hypertens..

[181] Zepeda R.J., Castillo R., Rodrigo R., Prieto J.C., Aramburu I., Brugere S., Galdames K., Noriega V., Miranda H.F. (2012). Effect of Carvedilol and Nebivolol on Oxidative Stress-Related Parameters and Endothelial Function in Patients with Essential Hypertension. Basic. Clin. Pharmacol. Toxicol..

[182] Kukin M.L., Kalman J., Charney R.H., Levy D.K., Buchholz-Varley C., Ocampo O.N., Eng C. (1999). Prospective, Randomized Comparison of Effect of Long-Term Treatment with Metoprolol or Carvedilol on Symptoms, Exercise, Ejection Fraction, and Oxidative Stress in Heart Failure. Circulation.

[183] Yao E.-H., Fukuda N., Matsumoto T., Katakawa M., Yamamoto C., Han Y., Ueno T., Kobayashi N., Matsumoto K. (2008). Effects of the Antioxidative Beta-Blocker Celiprolol on Endothelial Progenitor Cells in Hypertensive Rats. Am. J. Hypertens..

[184] Mehta J.L., Lopez L.M., Chen L., Cox O.E. (1994). Alterations in Nitric Oxide Synthase Activity, Superoxide Anion Generation, and Platelet Aggregation in Systemic Hypertension, and Effects of Celiprolol. Am. J. Cardiol..

[185] Okrucká A., Pechán J., Balazovjech I. (1993). The Effect of Short-Term Celiprolol Therapy on Platelet Function in Essential Hypertension. Cardiology.

[186] Kobayashi N., Mori Y., Nakano S., Tsubokou Y., Shirataki H., Matsuoka H. (2001). Celiprolol Stimulates Endothelial Nitric Oxide Synthase Expression and Improves Myocardial Remodeling in Deoxycorticosterone Acetate-Salt Hypertensive Rats. J. Hypertens..

[187] Cominacini L., Fratta Pasini A., Garbin U., Nava C., Davoli A., Criscuoli M., Crea A., Sawamura T., Lo Cascio V. (2003). Nebivolol and Its 4-Keto Derivative Increase Nitric Oxide in Endothelial Cells by Reducing Its Oxidative Inactivation. J. Am. Coll. Cardiol..

[188] Filion K.B., Azoulay L., Platt R.W., Dahl M., Dormuth C.R., Clemens K.K., Hu N., Paterson J.M., Targownik L., Turin T.C. (2016). A Multicenter Observational Study of Incretin-Based Drugs and Heart Failure. NEJM.

[189] Kramer C.K., Ye C., Campbell S., Retnakaran R. (2018). Comparison of New Glucose-Lowering Drugs on Risk of Heart Failure in Type 2 Diabetes: A Network Meta-Analysis. JACC Heart Fail..

[190] Timmers L., Henriques J.P.S., de Kleijn D.P.V., Devries J.H., Kemperman H., Steendijk P., Verlaan C.W.J., Kerver M., Piek J.J., Doevendans P.A. (2009). Exenatide Reduces Infarct Size and Improves Cardiac Function in a Porcine Model of Ischemia and Reperfusion Injury. J. Am. Coll. Cardiol..

[191] Hocher B., Sharkovska Y., Mark M., Klein T., Pfab T. (2013). The Novel DPP-4 Inhibitors Linagliptin and BI 14361 Reduce Infarct Size after Myocardial Ischemia/Reperfusion in Rats. Int. J. Cardiol..

[192] Andreadou I., Efentakis P., Balafas E., Togliatto G., Davos C.H., Varela A., Dimitriou C.A., Nikolaou P.-E., Maratou E., Lambadiari V. (2017). Empagliflozin Limits Myocardial Infarction in Vivo and Cell Death in Vitro: Role of STAT3, Mitochondria, and Redox Aspects. Front. Physiol..

[193] Oshima H., Miki T., Kuno A., Mizuno M., Sato T., Tanno M., Yano T., Nakata K., Kimura Y., Abe K. (2019). Empagliflozin, an SGLT2 Inhibitor, Reduced the Mortality Rate after Acute Myocardial Infarction with Modification of Cardiac Metabolomes and Antioxidants in Diabetic Rats. J. Pharmacol. Exp. Ther..

[194] Bonnet F., Scheen A.J. (2018). Effects of SGLT2 Inhibitors on Systemic and Tissue Low-Grade Inflammation: The Potential Contribution to Diabetes Complications and Cardiovascular Disease. Diabetes Metab..

[195] Atkin S.L., Katsiki N., Banach M., Mikhailidis D.P., Pirro M., Sahebkar A. (2017). Effect of Dipeptidyl Peptidase-4 Inhibitors on Circulating Tumor Necrosis Factor-α Concentrations: A Systematic Review and Meta-Analysis of Controlled Trials. J. Diabetes Complicat..

[196] Lee Y.-S., Jun H.-S. (2016). Anti-Inflammatory Effects of GLP-1-Based Therapies beyond Glucose Control. Mediat. Inflamm..

[197] Tahara A., Kurosaki E., Yokono M., Yamajuku D., Kihara R., Hayashizaki Y., Takasu T., Imamura M., Li Q., Tomiyama H. (2013). Effects of SGLT2 Selective Inhibitor Ipragliflozin on Hyperglycemia, Hyperlipidemia, Hepatic Steatosis, Oxidative Stress, Inflammation, and Obesity in Type 2 Diabetic Mice. Eur. J. Pharmacol..

[198] Tahara A., Kurosaki E., Yokono M., Yamajuku D., Kihara R., Hayashizaki Y., Takasu T., Imamura M., Li Q., Tomiyama H. (2014). Effects of Sodium-Glucose Cotransporter 2 Selective Inhibitor Ipragliflozin on Hyperglycaemia, Oxidative Stress, Inflammation and Liver Injury in Streptozotocin-Induced Type 1 Diabetic Rats. J. Pharm. Pharmacol..

[199] (1994). Randomised Trial of Cholesterol Lowering in 4444 Patients with Coronary Heart Disease: The Scandinavian Simvastatin Survival Study (4S). Lancet.

[200] Sacks F.M., Pfeffer M.A., Moye L.A., Rouleau J.L., Rutherford J.D., Cole T.G., Brown L., Warnica J.W., Arnold J.M., Wun C.C. (1996). The Effect of Pravastatin on Coronary Events after Myocardial Infarction in Patients with Average Cholesterol Levels. Cholesterol and Recurrent Events Trial Investigators. NEJM.

[201] (1998). Long-Term Intervention with Pravastatin in Ischaemic Disease (LIPID) Study Group Prevention of Cardiovascular Events and Death with Pravastatin in Patients with Coronary Heart Disease and a Broad Range of Initial Cholesterol Levels. NEJM.

[202] West of Scotland Coronary Prevention Study: Implications for Clinical Practice (1996). The WOSCOPS Study Group. Eur. Heart J..

[203] Downs J.R., Clearfield M., Weis S., Whitney E., Shapiro D.R., Beere P.A., Langendorfer A., Stein E.A., Kruyer W., Gotto A.M. (1998). Primary Prevention of Acute Coronary Events with Lovastatin in Men and Women with Average Cholesterol Levels: Results of AFCAPS/TexCAPS. Air Force/Texas Coronary Atherosclerosis Prevention Study. JAMA.

[204] (2002). Heart Protection Study Collaborative Group MRC/BHF Heart Protection Study of Cholesterol Lowering with Simvastatin in 20,536 High-Risk Individuals: A Randomised Placebo-Controlled Trial. Lancet.

[205] Liao J.K. (2005). Clinical Implications for Statin Pleiotropy. Curr. Opin. Lipidol..

[206] Liao J.K., Laufs U. (2005). Pleiotropic Effects of Statins. Annu. Rev. Pharmacol. Toxicol..

[207] Ridker P.M., Danielson E., Fonseca F.A.H., Genest J., Gotto A.M., Kastelein J.J.P., Koenig W., Libby P., Lorenzatti A.J., MacFadyen J.G. (2008). Rosuvastatin to Prevent Vascular Events in Men and Women with Elevated C-Reactive Protein. NEJM.

[208] Ridker P.M., Rifai N., Clearfield M., Downs J.R., Weis S.E., Miles J.S., Gotto A.M. (2001). Air Force/Texas Coronary Atherosclerosis Prevention Study Investigators Measurement of C-Reactive Protein for the Targeting of Statin Therapy in the Primary Prevention of Acute Coronary Events. NEJM.

[209] Ridker P.M., Rifai N., Pfeffer M.A., Sacks F., Braunwald E. (1999). Long-Term Effects of Pravastatin on Plasma Concentration of C-Reactive Protein. The Cholesterol and Recurrent Events (CARE) Investigators. Circulation.

[210] Zorov D.B., Juhaszova M., Sollott S.J. (2014). Mitochondrial Reactive Oxygen Species (ROS) and ROS-Induced ROS Release. Physiol. Rev..

[211] Sabbah H.N. (2016). Targeting Mitochondrial Dysfunction in the Treatment of Heart Failure. Expert Rev. Cardiovasc. Ther..

[212] Ni R., Cao T., Xiong S., Ma J., Fan G.-C., Lacefield J.C., Lu Y., Le Tissier S., Peng T. (2016). Therapeutic Inhibition of Mitochondrial Reactive Oxygen Species with Mito-TEMPO Reduces Diabetic Cardiomyopathy. Free Radic. Biol. Med..

[213] Dikalova A.E., Bikineyeva A.T., Budzyn K., Nazarewicz R.R., McCann L., Lewis W., Harrison D.G., Dikalov S.I. (2010). Therapeutic Targeting of Mitochondrial Superoxide in Hypertension. Circ. Res..

[214] Jeong E.-M., Chung J., Liu H., Go Y., Gladstein S., Farzaneh-Far A., Lewandowski E.D., Dudley S.C. (2016). Role of Mitochondrial Oxidative Stress in Glucose Tolerance, Insulin Resistance, and Cardiac Diastolic Dysfunction. J. Am. Heart Assoc..

[215] Jiménez-González S., Marín-Royo G., Jurado-López R., Bartolomé M.V., Romero-Miranda A., Luaces M., Islas F., Nieto M.L., Martínez-Martínez E., Cachofeiro V. (2020). The Crosstalk between Cardiac Lipotoxicity and Mitochondrial Oxidative Stress in the Cardiac Alterations in Diet-Induced Obesity in Rats. Cells.

[216] Kim S., Song J., Ernst P., Latimer M.N., Ha C.-M., Goh K.Y., Ma W., Rajasekaran N.-S., Zhang J., Liu X. (2020). MitoQ Regulates Redox-Related Noncoding RNAs to Preserve Mitochondrial Network Integrity in Pressure-Overload Heart Failure. Am. J. Physiol. Heart Circ. Physiol..

[217] Doughan A.K., Dikalov S.I. (2007). Mitochondrial Redox Cycling of Mitoquinone Leads to Superoxide Production and Cellular Apoptosis. Antioxid. Redox Signal..

[218] Pokrzywinski K.L., Biel T.G., Kryndushkin D., Rao V.A. (2016). Therapeutic Targeting of the Mitochondria Initiates Excessive Superoxide Production and Mitochondrial Depolarization Causing Decreased MtDNA Integrity. PLoS ONE.

[219] Gibbons J.J., Abraham R.T., Yu K. (2009). Mammalian Target of Rapamycin: Discovery of Rapamycin Reveals a Signaling Pathway Important for Normal and Cancer Cell Growth. Semin. Oncol..

[220] Yang X., Yang C., Farberman A., Rideout T.C., de Lange C.F.M., France J., Fan M.Z. (2008). The Mammalian Target of Rapamycin-Signaling Pathway in Regulating Metabolism and Growth. J. Anim. Sci..

[221] Sanches-Silva A., Testai L., Nabavi S.F., Battino M., Pandima Devi K., Tejada S., Sureda A., Xu S., Yousefi B., Majidinia M. (2020). Therapeutic Potential of Polyphenols in Cardiovascular Diseases: Regulation of MTOR Signaling Pathway. Pharmacol. Res..

[222] Sciarretta S., Forte M., Frati G., Sadoshima J. (2018). New Insights Into the Role of MTOR Signaling in the Cardiovascular System. Circ. Res..

[223] Zhao D., Yang J., Yang L. (2017). Insights for Oxidative Stress and MTOR Signaling in Myocardial Ischemia/Reperfusion Injury under Diabetes. Oxid. Med. Cell. Longev..

[224] Elloso M.M., Azrolan N., Sehgal S.N., Hsu P.-L., Phiel K.L., Kopec C.A., Basso M.D., Adelman S.J. (2003). Protective Effect of the Immunosuppressant Sirolimus against Aortic Atherosclerosis in Apo E-Deficient Mice. Am. J. Transplant..

[225] Castro C., Campistol J.M., Sancho D., Sánchez-Madrid F., Casals E., Andrés V. (2004). Rapamycin Attenuates Atherosclerosis Induced by Dietary Cholesterol in Apolipoprotein-Deficient Mice through a P27 Kip1 -Independent Pathway. Atherosclerosis.

[226] Chen W.Q., Zhong L., Zhang L., Ji X.P., Zhang M., Zhao Y.X., Zhang C., Zhang Y. (2009). Oral Rapamycin Attenuates Inflammation and Enhances Stability of Atherosclerotic Plaques in Rabbits Independent of Serum Lipid Levels. Br. J. Pharmacol..

[227] Kurdi A., Martinet W., De Meyer G.R.Y. (2018). MTOR Inhibition and Cardiovascular Diseases: Dyslipidemia and Atherosclerosis. Transplantation.

[228] Martinet W., De Loof H., De Meyer G.R.Y. (2014). MTOR Inhibition: A Promising Strategy for Stabilization of Atherosclerotic Plaques. Atherosclerosis.

[229] Xu X., Lu Z., Fassett J., Zhang P., Hu X., Liu X., Kwak D., Li J., Zhu G., Tao Y. (2014). Metformin Protects against Systolic Overload-Induced Heart Failure Independent of AMP-Activated Protein Kinase A2. Hypertension.

[230] Liu M., Wilk S.A., Wang A., Zhou L., Wang R.-H., Ogawa W., Deng C., Dong L.Q., Liu F. (2010). Resveratrol Inhibits MTOR Signaling by Promoting the Interaction between MTOR and DEPTOR. J. Biol. Chem..

[231] Demidenko Z.N., Blagosklonny M.V. (2009). At Concentrations That Inhibit MTOR, Resveratrol Suppresses Cellular Senescence. Cell Cycle.

[232] Brito P.M., Devillard R., Nègre-Salvayre A., Almeida L.M., Dinis T.C.P., Salvayre R., Augé N. (2009). Resveratrol Inhibits the MTOR Mitogenic Signaling Evoked by Oxidized LDL in Smooth Muscle Cells. Atherosclerosis.

[233] Song J., Huang Y., Zheng W., Yan J., Cheng M., Zhao R., Chen L., Hu C., Jia W. (2018). Resveratrol Reduces Intracellular Reactive Oxygen Species Levels by Inducing Autophagy through the AMPK-MTOR Pathway. Front. Med..

[234] Guo S., Long M., Li X., Zhu S., Zhang M., Yang Z. (2016). Curcumin Activates Autophagy and Attenuates Oxidative Damage in EA.Hy926 Cells via the Akt/MTOR Pathway. Mol. Med. Rep..

[235] Liu R., Zhang H.-B., Yang J., Wang J.-R., Liu J.-X., Li C.-L. (2018). Curcumin Alleviates Isoproterenol-Induced Cardiac Hypertrophy and Fibrosis through Inhibition of Autophagy and Activation of MTOR. Eur. Rev. Med. Pharmacol. Sci..

[236] Chen C., Zhou M., Ge Y., Wang X. (2020). SIRT1 and Aging Related Signaling Pathways. Mech. Ageing Dev..

